# Vascularization of neonatal liver lobules presages adult liver size

**DOI:** 10.1038/s41467-025-64930-w

**Published:** 2025-11-13

**Authors:** D. Berfin Azizoglu, Karina Perez, Sherry Li Zheng, Shahadat Rahman, Ellen Youngsoo Rim, Teni Anbarchian, Matt Fish, Kyle M. Loh, Kristy Red-Horse, Roel Nusse

**Affiliations:** 1 Howard Hughes Medical Institute, Department of Developmental Biology, Institute for Stem Cell Biology and Regenerative Medicine, Stanford University School of Medicine, Stanford, CA USA; 2 Department of Developmental Biology, Stanford Institute for Stem Cell Biology and Regenerative Medicine, Stanford University School of Medicine, Stanford, CA USA; 3 Howard Hughes Medical Institute, Department of Biology, Institute for Stem Cell Biology and Regenerative Medicine, Stanford University, Stanford, CA USA; 4 Present Address: Department of Cell Biology and Physiology, University of North Carolina, Chapel Hill, NC USA

**Keywords:** Organogenesis, Angiogenesis, Morphogen signalling

## Abstract

Organs vary in size between and within species to match organismal needs. Theoretical work has proposed that scaling of organs and body parts relies on energy-transport systems, the vascular system in mammals. Here, we use quantitative clonal mapping and volumetric imaging combined with novel molecular and genetic tools to identify temporal and spatial constraints that establish mouse liver size. We find that adult liver size is foreshadowed during a neonatal period when functional units, termed lobules, initiate growth. Nascent lobules are vascularized by prominent sprouting angiogenesis of the hepatic vein, restricted to the periphery of the organ. When Wnt signals are ablated in the single cell-layered mesothelium at the periphery, neonatal growth is disturbed, and the liver adopts a compromised size set point. Similarly, when venous angiogenesis is inhibited, nascent lobules remain small and the liver fails to reach proper size. In unperturbed animals, vein sprouting rapidly declines within a week after birth and well before hepatocyte division stops. These findings suggest that vascularization in the neonate assists in the determination of adult liver size. Together, these results lead us to propose a vasculature-centric experimental framework for studying organ size control and scaling in mammals.

## Abstract (web summary)

Here they identify a short-lived vascular endothelial progenitor pool that predominantly vascularizes the mouse liver during postnatal growth and contributes to liver size control.

## Introduction

Organ size scales with animal size in mammals that themselves vary up to a hundred million times in weight^1^. How mammalian organs reach their proper size across such a wide range of scales despite similar developmental trajectories is a fundamental question in biology with direct implications for regenerative medicine^2,3^.

Our understanding of organ size control has largely relied on studies of parenchymal cell proliferation and the relevant signaling networks^1^. However, parenchymal cells alone cannot support an organ; multiple cell types must integrate into anatomical units for an organ to form and function^4^. Indeed, one central regulator of parenchymal cell proliferation, Hippo signaling, fails to instruct architecturally normal organ growth on its own^5–8^. Theoretical modeling from the 1990s instead suggests that energy-distributing vascular systems are a key component of scaling body parts relative to the body^9^. These observations highlight the need to study organ size in the context of multicellular integration and, in particular, vascularization. Yet, experimental studies on whether or how vascularization helps determine organ size have lagged behind. The mammalian liver provides a unique experimental system to tackle this question, as liver size scales proportionally with high precision between individual animals^1,10,11^, offering a highly tractable model.

Here, we focus on the mouse liver to interrogate the multicellular-scale mechanisms that determine mammalian organ size. The adult liver consists of repeated functional units termed lobules^12^. Thus, proper liver size must be achieved by fine-tuning the size of lobules in a given individual. We investigate the mechanisms that establish liver size by studying how nascent liver lobules grow and vascularize, and, importantly, cease to grow. We use genetic lineage-tracing and quantitative clonal mapping of the vascular endothelial cells to identify actively growing, nascent lobules at the periphery of the postnatal liver. When we genetically ablate Wnt signaling from the liver mesothelium at the periphery, the liver fails to grow to the proper size. We find that the nascent lobules are dominantly vascularized by sprouting angiogenesis of the hepatic vein. When venous angiogenesis is genetically inhibited through Notch over-activation, lobules remain small and the liver fails to reach proper size. Finally, we show that venous angiogenesis rapidly declines within one week of birth, well before hepatocyte proliferation stops. This suggests that nascent lobule vascularization and growth are restricted to the neonatal period, foreshadowing final lobule size and liver size by adulthood. Our results propose lobule vascularization as an important, rate-limiting step in determining final liver size.

## Results

### Novel liver lobule marker reveals generation of lobules after birth

**Fig. 1:**
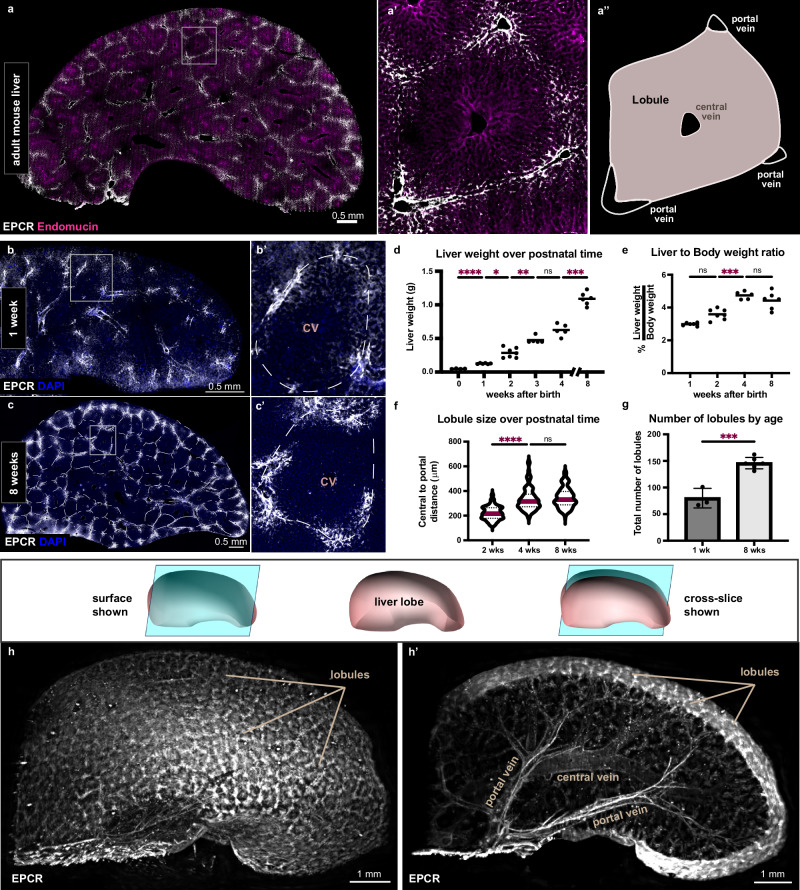
Novel liver lobule marker reveals generation of lobules after birth. **a–a**’, Thick section of the adult mouse liver immunostained for the lobule boundary marker EPCR and the venous endothelial marker Endomucin. **a**”, Schematic representation of the lobule. **b, c**’ Thick section of the liver immunostained for the lobule boundary marker EPCR from 1 week- and 8 week-old mice. Dotted lines delineate lobules. **d** Increase in absolute liver weight over postnatal time for *n* = 4 (newborn, male), *n* = 6 (1 week, male), *n* = 7 (2 weeks, male), *n* = 5 (3 weeks, male), *n* = 5 (4 weeks, male), and *n* = 6 (8 weeks, male) mice. Brown-Forsythe Anova test and Dunnett’s multiple comparisons test performed. For clarity, differences between consecutive ages are shown. Adjusted p-values from left to right: *<0.0001*, *0.0126, 0.0049, 0.1526, 0.0002*. **e** Liver-to-body weight ratio as percentage for *n* = 6 (1 week, male), *n* = 7 (2 weeks, male), *n* = 5 (4 weeks, male), and *n* = 6 (8 weeks, male) mice. Brown-Forsythe Anova test and Dunnett’s multiple comparisons test performed. For clarity, differences between consecutive ages are shown. Adjusted p-values from left to right: *0.0911, 0.0002, 0.3906*. **f** Violin plot shows the distance from the central vein to portal vessels at lobule boundaries in livers of 2 week-, 4 week-, and 8-week-old male mice. Red bars represent the mean. A total of *n* = 210 (2 weeks), *n* = 147 (4 weeks), and *n* = 162 (8 weeks) lobules were scored in 3 mice per group. Brown-Forsythe Anova test and Dunnett’s multiple comparisons test, adjusted p-values from left to right: *<0.0001*, > *0.9999*. **g** The total number of lobules in one whole liver lobe of 1 week-old (*n* = 3) and 8-week-old (*n* = 6) male mice. Mean values ± s.d. shown. Unpaired *t*-test, *p-value* = *0.0002*. **h–h**’, Volumetric images of whole liver lobes iDisco-cleared and immunostained for the lobule boundary marker EPCR are shown in surface view in **h** and slice view in **h**’. ns, not significant. DAPI in blue depicts nuclei. Source data are provided in the Fig. 1 Source Data file.

To study how lobule organization relates to liver size in mice, we sought to identify a molecular marker of mouse liver lobules. Demarcating liver lobules in rodents and humans has proven challenging, as the extracellular matrix components at lobule boundaries are scarce in these species, unlike in other mammals^12,13^. We found that a component of the coagulation pathway, endothelial protein C receptor (EPCR), is highly enriched in the portal veins and venules (referred to as portal vessels) and thus outlines liver lobules (Fig. 1a–a” and Suppl. Fig. 1a). To define when lobule boundaries are assembled, we assessed EPCR expression through the postnatal period (Fig. 1b, c’). Lobule boundaries were discernable as early as one week of age (Fig. 1b–b’ and Suppl. Fig. 1b–d’). These results establish EPCR as a novel marker of lobule boundaries and show that the mouse liver is organized into lobules over the neonatal period.

Next, we sought to define when the stereotyped ratio of liver weight to body weight, the liver size set point, is achieved. While the mouse liver grows in absolute weight from birth until eight weeks of age (Fig. 1d), we found that it reaches its stereotyped size set point well before that, at four weeks of age (Fig. 1e). This prompted us to examine how lobule size and numbers change over the same period. Lobules became larger over postnatal time (Fig. 1f and Suppl. Fig. 1e). Strikingly, lobule size was finalized by four weeks of age (Fig. 1f), long prior to the absolute liver weight, and concurrently with the liver-to-body weight ratio (Fig. 1e, f). These results couple lobule size to the liver size set point.

To determine potential changes in lobule numbers, we assessed the total number of lobules in whole liver lobes. To this end, we utilized iDisco clearing^14^ combined with EPCR immunostaining and light-sheet imaging (Fig. 1g, h’ and Supplementary Movies 1, 2). Lobules could be seen covering the entire surface of the imaged livers (Fig. 1h). EPCR labeling enabled us to simultaneously visualize the portal and central vein branches feeding into the lobules deep through the organ (Fig. 1h’). Unexpectedly, quantification of mature lobules revealed a twofold increase in lobule number over the postnatal period (Fig. 1g), indicating that close to half of adult liver lobules are in nascent form at birth and grow postnatally.

These findings suggest that the adult liver size set point is established postnatally through two mechanisms, lobule enlargement and nascent lobule growth.

### Cells divide and expand dramatically in lobules at the organ periphery

**Fig. 2:**
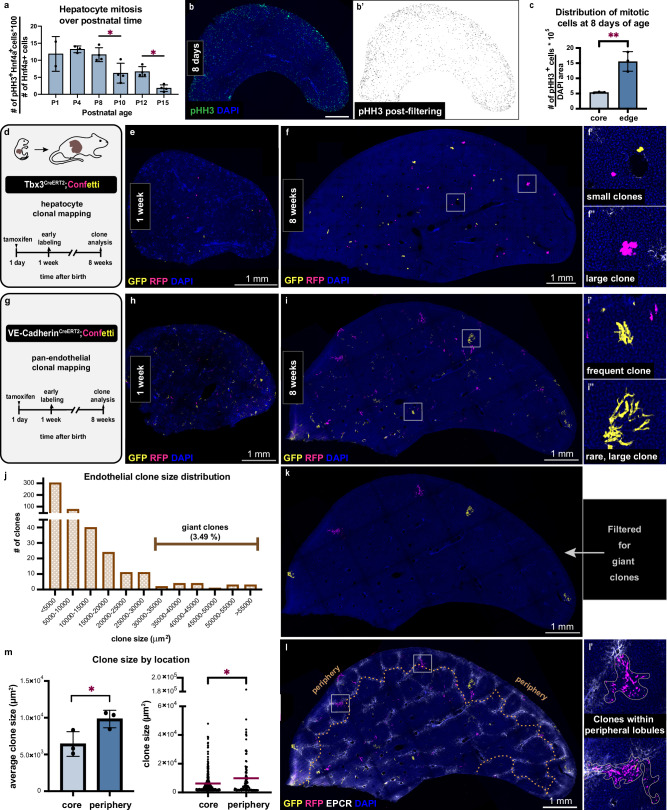
Cells expand dramatically in a subset of liver lobules at the organ periphery. **a** Quantification of mitotic hepatocytes by phospho-histone H3 (pHH3) and Hepatocyte nuclear factor 4a (Hnf4a) labeling at postnatal ages (P). Mean values ± s.d. *n* = 4 male mice per group, except *n* = 2 mice at P1 and *n* = 3 mice at P4. Ordinary one-way Anova analysis and Tukey’s multiple comparisons test performed. For clarity, significant differences between consecutive ages are shown. Adjusted p-values from left to right, including the non-significant comparisons between consecutive ages: *0.9749, 0.9050, 0.0189, 0.9996, 0.0398*. **b** Thick liver section at 8 days immunostained for pHH3. **b**’ Post-processing of image in (**b**) with intensity thresholding and particle analysis. **c** Quantification of pHH3+ cells at the edge and the core at 8 days from *nn*em> = 3 mice. Mean values ± s.d. Unpaired *t*-test, *p-value* = *0.0054*. **d** Schematic summary of the experimental design to map hepatocyte expansion. **e, f**” Tbx3^CreERT2^; Confetti thick liver section immunostained for GFP and RFP to visualize the early labeling at 1 week and the clones at 8 weeks. **g** Schematic summary of the experimental design to map endothelial cell expansion. **h, i**” VE-Cadherin^CreERT2^; Confetti thick liver section immunostained for GFP and RFP to visualize the early labeling at 1 week and the clones at 8 weeks. **j** Endothelial clone size distribution from VE-Cadherin^CreERT2^; Confetti livers at 8 weeks, *n* = 457 clones from 3 mice. **k** Clones in (**i**) filtered by size ( > 3 × 10^4^ µm^2^) to visualize only the giant clones. **l, l**’, Merge of image in (**i**) with EPCR immunostaining with the periphery delineated (dotted line). **m** Comparison of endothelial clone size between the core and the periphery in 8-week-old mice. Mean values ± s.d. per mouse (left) and individual clone values (right). *n* = 128 clones in the periphery and *n* = 329 clones in the core from 3 mice. Left: Ratio paired *t*-test, *p-value* = *0.0417*. Right: Welch’s *t*-test, *adjusted p-value* = *0.036*. DAPI in blue depicts nuclei. tam, tamoxifen. Source data are provided in the Fig. 2 Source Data file.

To understand what factors constrain liver size, we tracked the growth of nascent lobules in the postnatal liver. We reasoned that growing a lobule from its beginnings would require substantial cell proliferation. To survey the extent of proliferation, we measured the frequency of mitoses in full liver sections by phospho-histone H3 (pHH3) labeling. This analysis indeed identified high levels of hepatocyte division in the neonate (Fig. 2a and Suppl. Fig. 2a, b’). Spatial distribution of mitotic cells in the neonate was surprisingly uneven, with a strong enrichment towards the edge of the liver (Fig. 2b, c). This indicated that more cell division occurs at the edge of the neonatal liver.

We asked whether the uneven distribution of cell division translates to biased growth over the postnatal period. We monitored the long-term expansion of cells in the liver for hepatocytes and cells of the hepatic vasculature. To assess hepatocyte expansion, we performed lineage tracing using an inducible Tbx3^Confetti^ mouse model where Tbx3^CreERT2^ drives the Confetti reporter upon Tamoxifen administration (Fig. 2d). Tbx3 is neonatally expressed in many hepatocytes except those in the periportal zone^15^ (Suppl. Fig. 1a). We sparsely labeled the cells at one day of age and confirmed that, by one week of age, hepatocytes were labeled across the liver (Fig. 2e). By adulthood, many hepatocytes had formed multicellular clones (Fig. 2f). The clones were distributed across the liver, suggesting that growth occurred across the organ. However, we noted that particularly large clones had emerged towards the edge of the liver (Fig. 2f–f” and Suppl. Fig. 2c). Labeling of hepatocytes using the previously established R26^CreERT2^, ^16,17^ (R26^Confetti^) similarly revealed larger clones towards the edge of the liver (Suppl. Fig. 2d–f).

We next assessed the expansion of another major liver cell type, vascular endothelial cells, as vascularization is indispensable for the growth of the organ^18–21^. We targeted endothelial cells across vessel subtypes, i.e., veins, arteries, and capillaries, by combining the tamoxifen-inducible pan-endothelial VE-Cadherin^CreERT2^ with the Confetti reporter system (VeCad^Confetti^). We sparsely labeled the cells at one day of age and performed clonal analysis at adulthood (Fig. 2g). Labeled endothelial cells were detectable throughout the liver one week following tamoxifen induction (Fig. 2h), indicating that labeling occurs efficiently across the organ. By adulthood, most single cells expanded into multicellular clones (Fig. 2i–i”). Similar to hepatocytes, endothelial cells expanded across the liver, but larger endothelial clones appeared towards the edge (Fig. 2i–i”). Intriguingly, rare clones over 30,000 µm^2^ in area emerged, which we refer to as giant clones (Fig. 2j). These giant clones were strictly localized towards the edge of the liver (Fig. 2k).

The co-occurrence of larger endothelial and hepatocyte clones suggested accelerated growth at the edge of the organ. To test if this underpins nascent lobule growth, we mapped the endothelial clones against EPCR-marked lobule boundaries. This uncovered a striking relationship: the giant clones were 4 times more likely to reside within the outermost lobules at the edge of the liver, referred to as the periphery (Fig. 2l–l’ and Suppl. Fig. 2g). We confirmed that this is not due to differences in labeling efficiency (Suppl. Fig. 2h). Endothelial and hepatocyte clones in the periphery defined by lobule boundaries were significantly larger on average compared to the clones in the core (Fig. 2m and Suppl. Fig. 2c, f). Similarly, endothelial EdU incorporation was significantly higher at the periphery of the postnatal liver (Suppl. Fig. 2i, j). These results demonstrate that peripheral lobules grow extensively and are likely the youngest lobules.

Considering the increase in mature lobule number over the postnatal period, these findings suggest that the peripheral liver lobules initiate growth after birth.

### Mesothelial Wnts in the neonate fine-tune adult liver size

**Fig. 3:**
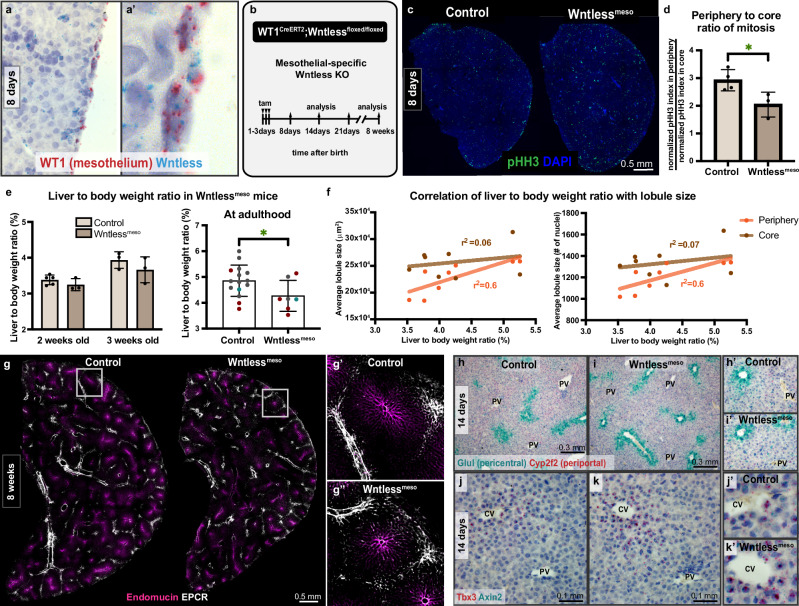
Liver size set point relies on peripheral tissue expansion promoted by mesothelial Wnt signaling in the neonate. **a–a**’, In situ hybridization on a representative wildtype liver section for *Wntless* and the mesothelial-specific *Wilms Tumor 1 (WT1)* at 8 days. **b** Schematic summary of the experimental design to determine the role of mesothelial Wnt signaling in postnatal growth and size control of the liver. tam, tamoxifen. **c** Thick left lobe liver sections of control and mesothelial-specific Wntless knock-out, or Wntless^meso^, mice at 8 days old, immunostained for phospho-histone H3. **d** Quantification of mitosis enrichment in the liver periphery for 8 days old control (*n* = 4) and Wntless^meso^ (*n* = 3) mice measured as the ratio of mitotic index in the periphery to that in the core. Mean values ± s.d. Unpaired *t*-test, *p-value* = *0.0376*. **e** Left, Liver-to-body weight ratio of 2-week-old male control (*n* = 5), 2-week-old male Wntless^meso^ (*n* = 3), 3-week-old male control (*n* = 3), and 3-week-old male Wntless^meso^ (*n* = 3) mice. Right, Liver-to-body weight ratio of 8-week-old male control (*n* = 15) and Wntless^meso^ (*n* = 7) mice, color-coded by litter. Mean values ± s.d. Unpaired *t*-test, *p-value* = *0.0464*. **f** Correlation between liver-to-body weight ratio and lobule size is shown for lobules in the core and at the periphery. Lobule size measured as absolute lobule area (left) and the number of nuclei per lobule (right). Data plotted from 8-week-old male control (*n* = 4) and Wntless^meso^ (*n* = 4) mice. Two-tailed Pearson correlation with 95% confidence interval used, *p value for periphery* = *0.02*, *for core* = *0.56*. **g–g**” Thick liver sections of male control and Wntless^meso^ mice immunostained for the venous marker Endomucin and the lobule boundary marker EPCR. **g**’ and **g**” show single lobules from each genotype. **h–k**’ In situ hybridization on liver sections from 14-day-old male control and Wntless^meso^ mice for pericentrally expressed *Glul* combined with periportally expressed *Cyp2f2* in (**h, i**’) and *Tbx3* combined with Axin2 in **j**, **k**’. **h**’–**k**’ show high magnification from respective low magnification images annotated with the matching letter. CV, central vein. PV, portal vessel. Source data are provided in the Fig. 3 Source Data file.

We hypothesized that the liver periphery must contain a source of growth signals to promote the development of nascent lobules. A single epithelial layer, the mesothelium, encapsulates the liver at its periphery and is a known source of mitogens in the embryo^22–24^. Among these mitogens, Wnts are highly expressed in the mesothelium^22^ and are well-known regulators of liver growth^25^. Indeed, the postnatal hepatic mesothelium expressed several Wnt ligands (Suppl. Fig. 3a–c) and Wntless required for Wnt secretion (Fig. 3a–a’), motivating us to test the role of mesothelial Wnt signals.

We generated mice with mesothelial-specific, inducible Wntless loss of function, WT1^CreERT2^; Wntless^f/f^ (Wntless^meso^) (Fig. 3b and Suppl. Fig. 3d–j). Depletion of mesothelial Wntless in neonates was sufficient to dampen the peripheral enrichment of mitosis (Fig. 3c, d and Suppl. Fig. 3k). While mitotic activity in control livers was concentrated to the periphery, mesothelial Wntless deletion led to a more even distribution of mitotic cells across the liver. This was accompanied by a progressive decline in liver size set point relative to controls (Fig. 3e). In adult Wntless^meso^ males, the liver-to-body weight ratio was significantly lower (Fig. 3e and Suppl. Fig. 3l–n). Wntless^meso^ mice and littermates showed considerable variability in liver-to-body weight ratios (Fig. 3e), presenting an opportunity to test whether liver size correlates with lobule size in individual mice. We divided the lobules into those at the periphery and in the core, and asked how the average lobule size in either location correlates with liver-to-body weight ratio. This analysis revealed a strong correlation of liver-to-body weight ratio with lobule size at the periphery (Fig. 3f). In striking contrast, lobule size in the core showed no correlation with liver-to-body weight ratio (Fig. 3f). These results suggest that nascent lobule growth at the periphery, and not lobule enlargement in the core, is a determinant of the liver size set point.

Wntless^meso^ livers displayed grossly normal architecture (Fig. 3g–g”), biliary organization (Suppl. Fig. 3o, p), and zonation (Fig. 3h–k’ and Suppl. Fig. 3q, r) in addition to normal lobule numbers (Suppl. Fig. 3s), uncoupling growth control from organization and zonation in the liver. To test whether reduced liver size in Wntless^meso^ was due to defective growth initiation in the neonate, we induced Wntless^meso^ ablation after the neonatal period is complete. In contrast with early Wntless ablation, late Wntless^meso^ mice had comparable liver-to-body weight ratios to controls (Suppl. Fig. 3t). These data supported a role for mesothelial Wnt signals specifically in the neonatal liver, followed by an impact on final liver size.

Together, these results identify the mesothelium as an unexpected organizer of liver growth and demonstrate that mesothelial Wnts contribute to liver size determination. Importantly, these findings reveal peripheral lobule growth as a determinant of the liver size set point.

### Peripheral vascularization of venous origin demarcates newly formed lobules

**Fig. 4:**
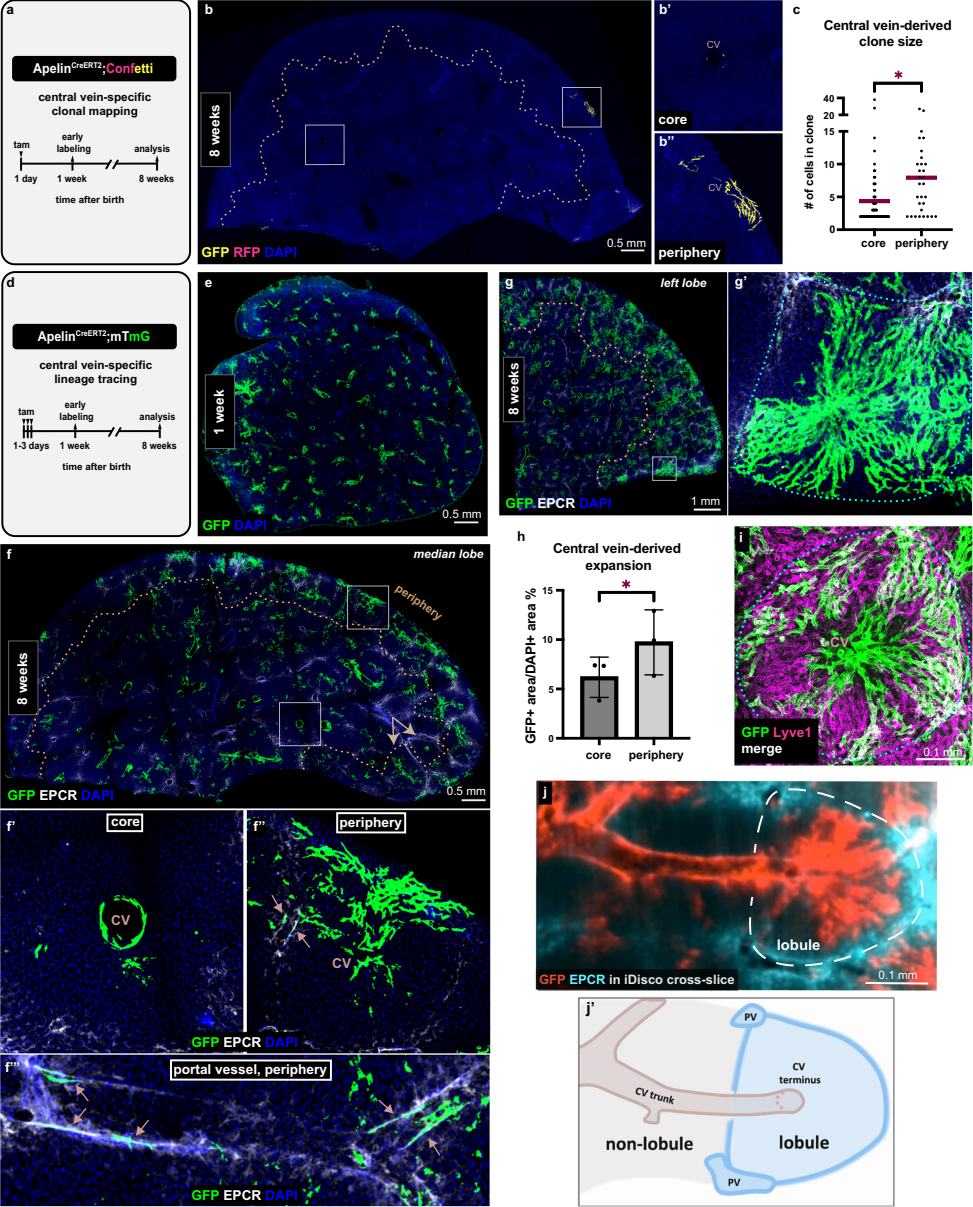
Central vein is a primary source of nascent lobule vasculature at the periphery of the liver. **a** Schematic summary of the experimental design to assess central vein-derived clonal expansion. **b–b**”, Apelin^CreERT2^; Confetti thick liver section immunostained for GFP and RFP to mark the clones at 8 weeks. The dotted line delineates the periphery. **c** Quantification of Apelin^CreERT2^; Confetti clone size measured as the number of cells per clone in the periphery compared to the core in 8-week-old mice. *n* = 70 clones in the core and *n* = 30 clones in the periphery from 3 mice were scored. Red bars represent the mean values. Welch’s *t*-test, *p-value* = *0.0108*. **d** Schematic summary of the experimental design to assess central vein-specific lineage tracing. **e** Apelin^CreERT2^;mTmG thick liver section immunostained for GFP to visualize the early labeling at 1 week. **f–f**”’, Apelin^CreERT2^;mTmG thick liver section immunostained for GFP and EPCR to visualize the tracing at 8 weeks. Dotted line delineates the periphery. Arrows in (**f**) show two neighboring lobules with dramatically different amounts of expansion. Arrows in (**f**”) and (**f**”’) show labeled cells within portal veins. **g–g**’ Apelin^CreERT2^;mTmG thick liver section taken from the left lobe immunostained for GFP and EPCR at 8 weeks. Dotted line in (**g**’) delineates the lobule boundaries. **h** Quantification of central vein-derived expansion in 8-week-old Apelin^CreERT2^;mTmG livers in the periphery compared to the core. *n* = 3 mice, mean values ± s.d. Ratio paired t test, *p-value* = *0.0290*. **i** Representative Apelin^CreERT2^;mTmG thick liver section immunostained for GFP and the sinusoidal marker Lyve1 to visualize the outcome of tracing in sinusoids. Dotted line delineates the lobule boundaries. **j** Cross-slice of iDisco-cleared Apelin^CreERT2^;mTmG whole lobe immunostained for GFP and EPCR. Dotted line delineates lobule boundaries. **j**’ Cartoon representation of lobule and non-lobule arrangements. Central vein lineage tracing outcome is not represented for clarity. DAPI in blue depicts nuclei. tam, tamoxifen. CV, central vein. PV, portal vessel. Source data are provided in the Fig. 4 Source Data file.

Consistent with nascent lobule growth and biased endothelial expansion at the periphery, we found the angiogenic cue Vegf-c^26^ concentrated in and near the liver mesothelium (Suppl. Fig. 3u, v). These observations prompted us to ask how nascent lobules become vascularized. Vascularization is likely to reveal key aspects of lobule formation, as blood vessels provide reliable landmarks in the lobule^12,13^. To study lobule vascularization, we first needed to gain genetic access to hepatic vessel subtypes. Thus, we surveyed a series of tamoxifen-inducible vascular endothelial Cre mouse lines (Suppl. Fig. 4). Among these, two lines were successful at targeting the large vessels of the lobule: Apelin^CreERT2^ that labeled the central veins (Suppl. Fig. 4b), and Efnb2^CreERT2^ that labeled the portal vessels (Suppl. Fig. 4c). By crossing these lines into the Confetti or the mTmG reporter, we induced labeling soon after birth (Fig. 4a, d) and assessed the contribution of each vessel type to the vascularization of nascent lobules at the periphery.

Endothelial cells of portal vessels formed clones as seen with Efnb2^Confetti^ tracing (Suppl. Fig. 5a–c); these clones were similarly sized and not spatially biased (Suppl. Fig. 5b’, c). By contrast, central vein endothelial cells marked by Apelin^Confetti^ formed clones of highly varying sizes (Fig. 4b, b”), evidently larger in the periphery than in the core (Fig. 4c and Suppl. Fig. 5d). These data demonstrated that central vein cells expand preferentially at the periphery, raising the possibility that central veins serve as origins of vasculature in nascent lobules.

To test whether central veins indeed vascularize nascent lobules, we labeled all central veins in the neonate using the mTmG reporter (Apelin^mTmG^) and traced the vein compartment as a whole until adulthood (Fig. 4d, and Suppl. Fig. 5c). Remarkably, endothelial cells sprouted and expanded out from the vein into the surrounding lobule in most lobules (79%) (Fig. 4e–g and Suppl. Fig. 5f, g). Central vein cell expansion was most pronounced in peripheral lobules; here, the progeny frequently reached the lobule boundaries (Fig. 4f’-h). Vein-derived progeny also reached and contributed to the portal vessels (Fig. 4f”-f”’, arrows). Since the lobule vascular bed is mainly composed of sinusoids^13,27^, these results implied that the sprouting vein cells differentiate into sinusoids. To determine whether that is the case, we co-labeled Apelin^mTmG^ livers with the hepatic sinusoid marker Lyve1^27^. Indeed, the vein cell progeny overlapped with sinusoids (Fig. 4i). These results identify the central vein as a vascularization source in nascent lobules.

Interestingly, each lobule was vascularized as an independent unit. Every Apelin^Confetti^ clone examined was entirely contained within the same lobule as the vein of origin and did not cross over to neighboring lobules (Fig. 4b, b” and Suppl. Fig. 5g). Furthermore, the extent of expansion was independent in each lobule. In two neighboring lobules, for instance, the vein of one lobule exhibited major expansion while the vein of the other expanded minimally (Fig. 4f, arrows). These data indicate that central vein cells sense lobule boundaries as they expand and solely vascularize their respective lobule. These findings suggest that central vein cell expansion can serve as a proxy for growth in a lobule.

If vein cell expansion is a suitable proxy for lobule growth, the expansion should be restricted to lobules and not occur in regions of the organ without proper lobule arrangement. To test this idea, we performed iDisco and whole lobe imaging of Apelin^mTmG^ livers (Supplementary Movie 3). Volumetric imaging revealed vein cell expansion throughout the liver, distinguishing the regions with and without lobule arrangement. In lobules, the central vein terminus was seen crossing through the center, with cross-sections of portal vessels at the boundaries (Fig. 4j–j’). By contrast, in regions lacking lobule arrangement, central vein trunks were seen running longitudinally (Fig. 4j–j’). Strikingly, the expansion from central veins was restricted to lobules and did not occur in the non-lobule regions (Fig. 4j–j’). These data reinforce the suitability of central vein cell expansion as a proxy for lobule growth.

To rule out potentially rare but significant expansion from portal vessels that could contribute to new lobules, we also performed lineage-tracing of portal vessels using Efnb2^mTmG^. This was particularly important given the low frequency of clone induction in Efnb2^Confetti^ mice (see “Methods”). Efnb2^mTmG^ tracing studies showed minimal expansion of the cells out of their original portal vessel compartment and no biased expansion in peripheral lobules (Suppl. Fig. 5h–l’). Thus, central veins, and not portal vessels, serve as a source of vascular beds in newly forming nascent lobules. These results establish central vein cells as a source of vascularization and venous angiogenesis as a proxy for nascent lobule growth.

### Nascent lobule growth at the periphery requires venous angiogenesis

**Fig. 5:**
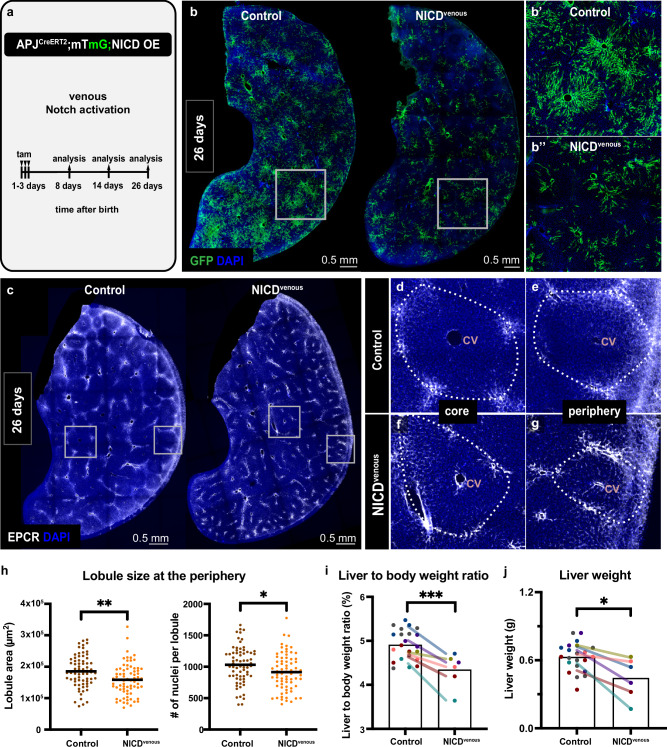
Venous angiogenesis is required for nascent lobule growth and proper liver size. **a** Schematic summary of the experimental design to assess the impact of Notch intracellular domain overexpression in the venous endothelium. **b–b**” APJ^CreERT2^;mTmG (control) and APJ^CreERT2^;mTmG;NICD (NICD^venous^) male thick liver sections immunostained for GFP at 26 days. **c–g**, APJ^CreERT2^;mTmG (control) and APJ^CreERT2^;mTmG;NICD (NICD^venous^) male thick liver sections immunostained for EPCR at 26 days. Dotted lines in (**d–g**) delineate the lobule boundaries. **h** Quantification of lobule size measured as absolute lobule area (left) and the number of nuclei per lobule (right) in APJ^CreERT2^;mTmG;NICD (NICD^venous^, *n* = 5) and littermate control (*n* = 5) males at 26 days at the periphery of the liver. Each dot represents a lobule. Black bars represent mean values. Unpaired *t*-test, *p-value* = *0.0023 (left) and 0.0176 (right)*. **i, j** Liver-to-body weight ratio and liver weight of 26-day-old male APJ^CreERT2^;mTmG;NICD (NICD^venous^, *n* = 6) and littermate control (*n* = 22) mice. Mice are color-coded by litter, and color-matched lines mark the difference between average values of control and NICD^venous^ within each litter. Two-way Anova and Tukey’s multiple comparisons test were performed. Adjusted *p-value for liver-to-body weight ratio* = *0.0003, for liver weight* = *0.0013*. tam, tamoxifen. CV, central vein. Source data are provided in the Fig. 5 Source Data file.

Our findings led us to hypothesize that vein-derived vascularization is required for nascent lobule growth. We next sought to test this idea by genetically inhibiting venous angiogenesis. We took advantage of the Notch intracellular domain (NICD) overexpression system^28^ that has been shown to hamper sprouting angiogenesis in multiple tissues^29–32^, and targeted this system to central veins and venules using the APJ^CreERT2^ mouse line^33^ (Fig. 5a and Suppl. Fig. 4e). We chose this line over Apelin^CreERT2^ as it evaded mosaicism caused by X-linkage and left the Apelin locus intact (see Methods). As an added advantage, the use of APJ^CreERT2^ maximized coverage of venule endothelial cells (Suppl. Fig. 4b, e) that would otherwise sprout out from the vein and continue to expand (Fig. 4b”, f”) before Tamoxifen induction completes in pups.

APJ^CreERT2^;mTmG livers exhibited considerable label coverage by weaning age (Fig. 5b, left), confirming the prevalence of venous angiogenesis in the postnatal liver. Upon induction of ectopic Notch activity in the venous endothelium in APJ^CreERT2^;NICD;mTmG (NICD^venous^) mice, angiogenesis was hampered (Fig. 5a, b”). These results indicated that the venous endothelium depends on a Notch-low state to undergo sprouting, as established in other tissues^29–32^. We then asked how hampered venous angiogenesis impacts lobule growth. Analysis of lobules at the periphery revealed that NICD^venous^ livers fail to grow nascent lobules to proper size (Fig. 5c–h). In stark contrast, lobules in the core of NICD^venous^ livers were larger on average (Suppl. Fig. 6a), suggesting no requirement of venous angiogenesis, and hinting at potential compensatory growth in the core. These data indicated that veins and venules vascularize nascent lobules at the periphery, but not lobules in the core, despite ongoing lobule enlargement. Indeed, NICD^venous^ livers were comparable to controls in size at early postnatal ages (Suppl. Fig. 6b). However, by weaning age, NICD^venous^ liver growth lagged behind, with a disrupted liver-to-body weight ratio in males (Fig. 5i, j and Suppl. Fig. 6c–k). NICD^venous^ animals displayed high mortality with increasing age and did not survive to adulthood (Suppl. Fig. 6l). This is in line with insufficient liver growth and the presence of other abnormalities, including cardiac hypertrophy and overall stunted growth in NICD^venous^ mice. Endothelial Notch overexpression in the liver has previously been shown to reduce Wnt expression^34,35^, which might underlie reduced liver size. We tested whether the lag in NICD^venous^ liver growth could be explained by reduced Wnt signals as opposed to hampered angiogenesis. NICD^venous^ animals exhibited endothelial Wnt expression comparable to controls (Suppl. Fig. 6m), arguing against Wnt-dependent liver size reduction in these mice. Importantly, lobule number was unaffected in NICD^venous^ livers (Suppl. Fig. 6n), indicating that lobule number is set independent of postnatal venous angiogenesis, likely before birth.

Together, these findings are in line with the notion that venous angiogenesis helps establish proper liver size through vascularization of nascent lobules. While we cannot definitively conclude that disrupted liver growth in NICD^venous^ mice is due to hampered venous angiogenesis, our results support a distinctive role for venous angiogenesis in vascularizing newly formed liver lobules.

### Central vein cell sprouting declines rapidly during the neonatal period

Our findings suggested that the extent of nascent lobule growth determines final liver size (Fig. 3f). This prompted us to ask when nascent lobules cease to grow and limit liver size. To identify when nascent lobule growth terminates, we monitored central vein cell sprouting.

**Fig. 6:**
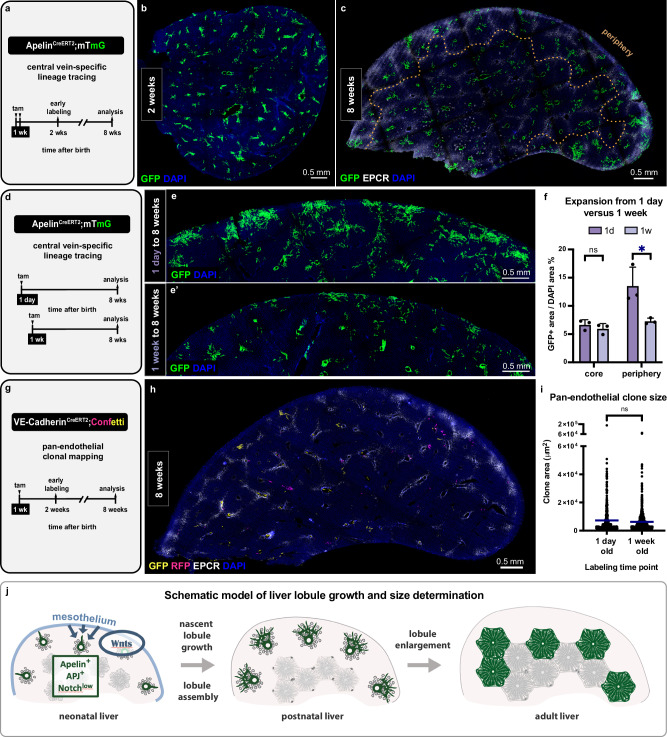
Nascent lobule vascularization declines neonatally. **a** Schematic summary of the experimental design to assess central vein lineage tracing induced at 1 week (injections on postnatal days 6 and 7). **b** Apelin^CreERT2^;mTmG thick liver section immunostained for GFP to visualize the early labeling at 2 weeks. **c** Apelin^CreERT2^;mTmG thick liver section immunostained for GFP and EPCR to visualize the tracing from 1 week to 8 weeks. The dotted line delineates the periphery. **d** Schematic summary of the experimental design to compare central vein lineage tracing induced at 1 day (injections at postnatal days 1–3) versus 1 week (injections on postnatal days 6 and 7). **e–e**’, The periphery of Apelin^CreERT2^;mTmG livers lineage traced from 1 day-to-8 weeks in (**e**) and 1 week-to-8 weeks in (**e**’) immunostained for GFP. **f** Quantification of central vein-derived expansion from 1 day-to-8 weeks compared to 1 week-to-8 weeks at the periphery and in the core. *n* = 3 males per group, mean values ± s.d. Two-way Anova and Tukey’s multiple comparisons test performed. Adjusted *p-value for core* = *0.9612, for periphery* = *0.0116*. **g** Schematic summary of the experimental design to map endothelial cell expansion from 1 week on. **h** VE-Cadherin^CreERT2^; Confetti thick liver section immunostained for GFP, RFP, and EPCR to spatially map the clones that formed from 1 week to 8 weeks. **i** Comparison of endothelial clone size in 8-week-old mice that were traced from 1 day versus from 1 week. *n* = 457 clones from 3 mice (labeled at 1 day) and *n* = 702 clones from 3 mice (labeled at 1 week). Blue bars represent mean values. Welch’s *t*-test, *p-value* = *0.0909*. **j** Schematic model of multicellular scale mechanisms that determine liver size. DAPI in blue depicts nuclei. ns, not significant; tam, tamoxifen. Source data are provided in the Fig. 6 Source Data file.

We labeled central vein cells in one-day versus one-week-old pups and compared their respective expansion by adulthood (Fig. 6a–f). This revealed a striking difference. When labeled at one week of age, central vein cells expanded minimally by adulthood, occupying only a small fraction of the periphery (Fig. 6c). This was in stark contrast with widespread expansion in peripheral lobules when central vein cells were labeled at one day of age (Fig. 6e, f). This difference may be caused either by major cellular expansion within the first week itself or a decline in the initial sprouting of the cells by one week of age. We assessed cellular expansion within the first week and found it to be minimal (Suppl. Fig. 4b). Interestingly, the expression of Apelin in the central vein endothelium followed a similar trajectory. Apelin was expressed at high levels in central vein endothelial cells at neonatal ages and was virtually absent in later life (Suppl. Fig. 7a), consistent with its pro-angiogenic nature^36–40^. These results together identify a rapid decline in the initial sprouting of central vein cells within the first postnatal week.

We next sought to determine whether the drastic temporal change in sprouting is specific to vein endothelial cells, as opposed to occurring across endothelia. We performed Vecad^Confetti^ labeling in one-week-old pups and asked how the clonal expansion by adulthood compares to expansion from one day of age in bulk endothelial cells. We found that the average clone size as well as the clone size distribution are largely similar between those monitored from 1 day versus 1 week of age (Fig. 6g–i and Suppl. Fig. 7b–d). Thus, the majority of endothelial cells do not exhibit a drastic change in expansion behavior over the first postnatal week, and the rapid decline in sprouting is unique to central vein cells.

These findings suggest that the progenitor behavior of central vein cells is restricted to the neonatal period. Meanwhile, the progeny of central vein cells continues to expand more at the periphery after the neonatal period (Suppl. Fig. 7e). This is consistent with continued vascularization and enlargement of the peripheral lobules over the extended postnatal period.

Together, these results identify a bottleneck for nascent lobule growth within the neonatal week. Notably, this timing precedes the decline of cell proliferation in the postnatal liver^41^ (Fig. 2a). Thus, vein cell sprouting emerges as a limiting factor in nascent lobule growth and in determining final liver size.

## Discussion

Our results identify five steps of multicellular integration that presage the mouse liver size. At birth, the liver is immature with no recognizable lobules (Fig. 6j). Over the first week after birth, the liver tissue is assembled into lobules (*lobule assembly*). Meanwhile, nascent lobules initiate primary growth at the organ edge, vascularized by sprouting at central vein endings (*nascent lobule growth*). Newly growing lobules obey pre-existing lobule boundaries. By the second week, vascular progenitors stop sprouting, and nascent lobules terminate primary growth, setting a limit on final liver size (*conclusion of nascent lobule growth*). Lobules at the edge continue to grow through proliferation of hepatocytes and endothelial cells (*lobule enlargement)*, until all lobules reach proper size by weaning age (*conclusion of lobule enlargement*). At weaning, lobule size is finalized, and the organ reaches its stereotyped size in ratio to the body (Fig. 6j).

These findings extend previous work that has defined the neonatal period as the endpoint of functional unit addition in other rodent organs^42,43^. We therefore propose that the neonatal period is a decisive period for rodent organ size control. Our results identify growth at the periphery of the liver as a limiting factor on adult mouse liver size, extending previous findings in vertebrates^44,45^. While the adult liver alters its size in response to perturbations^46^, our findings suggest that liver size is pre-constrained during development by determinants of lobule growth. Future research will be necessary to study in depth how nascent lobule growth co-occurs with other processes, such as bile duct development and zonation^41,47^. We find that the single cell-layered mesothelium encasing the liver spatially organizes growth and ensures that the liver will reach its stereotyped size. The mesothelium encapsulates most vertebrate internal organs^48^, warranting further investigation into the role of this cell layer in organ growth and size control. At the center of this work, our results identify the hepatic vein as a required in vivo vascular source for the growth of nascent functional units in the mouse liver. Future Notch-independent manipulations will be invaluable to further interrogate the role of hepatic vein angiogenesis. Notably, we find that the process of size determination in the liver is sex-dependent, motivating future studies on sex-specific mechanisms. While our work focused on the postnatal growth period, the regenerating liver must similarly engage size control. Our results motivate future research into the mechanisms of vascularization and lobule growth during liver regeneration. Together with previous work^49–52^, our findings contribute to a foundation for building vascularized liver functional units with bona fide organization and size for regenerative medicine applications.

Finally, we demonstrate that the vascular source of new functional units is lost prior to the decline of hepatic cell proliferation in the liver. This implies that liver growth is limited by expansion of the vascular network rather than hepatic cell proliferation. Theoretical studies have previously proposed vascular geometry as a key determinant of allometric scaling in biology^9^. According to this model, the scaling of body parts with organism size can be explained by the features of branching transport systems, the vascular system in mammals. Our experimental results advance this theoretical work and lead us to postulate that temporally limited vascular expansion in mice helps scale liver size. Overall, this study provides a unique framework that we expect will guide future investigations into organ growth and size control.

## Methods

### Mice

The Institutional Animal Care and Use Committee at Stanford University approved all animal methods and experiments. Animal experiments were performed with mouse pups and adult male and female 8-week-old mice. Data from males and females were pooled unless otherwise noted below or in the figure legends. Weaning age was set at 22–25 days. All mice were housed under a 12 h:12 h light:dark cycle at 22–25 °C and 30–70% humidity. Mice received rodent chow and water ad libitum. C56BL/6 mice were used for wild-type studies. *mTmG* (007676), *Confetti* (017492), *R26* ^*CreERT2*^ (008463), *WT1*^*CreERT2*^ (010912), and NICD (008159) mice were purchased from The Jackson Laboratory and bred in-house. *Tbx3*^*CreERT2*^ was generated by our lab^53^. *VE-Cadherin*^*CreERT2*^, also called *Cdh5-PAC-CreERT2 or* Cdh5-cre/ERT2^1Rha^, mice were generated by the Adams Lab^54^. *Wntless*^*flox*^ mice were a gift from Alan Cheng and were generated by the Lang Lab^55^. *Apelin*^*CreERT2*^ mice were generated by the Zhou lab^56^ and have been used as an invaluable tool to target sprouting endothelial cells^36–40^, in line with the identification of Apelin as an angiogenic factor and marker^57–59^. *Lyve1*^*CreERT2*^ mice were a gift from Darci Fink and were generated by the Tempero Lab^60^. *Esm1*^*CreERT2*^ mice were a gift and were generated by the Adams Lab^61^. *APJ*^*CreERT2*^ mice were generated by the Red-Horse lab^33^. *Efnb2*^*CreERT2*^ mice were generated by the Loh lab^62^.

CreERT2 mice were crossed into mTmG or Confetti mice for lineage tracing and clonal mapping studies. To study Wntless loss-of-function in the mesothelium, WT1^CreERT2^ mice were first crossed into Wntless^flox/flox^ mice. Then, WT1^CreERT2^; Wntless^flox/+^ or WT1^CreERT2^;Wntless^flox/flox^ mice were crossed into Wntless^flox/flox^ mice. Wntless^flox/+^ and Wntless^flox/flox^ littermates were used as controls. For *Apelin*^*CreERT2*^ lineage tracing studies, only males were used in order to avoid mosaic X chromosome inactivation, as the *Apelin* gene is X-linked.

### Tamoxifen treatment and lineage-tracing studies

Pups were induced with Tamoxifen by intragastric injection before postnatal day 3 and by intraperitoneal injection afterwards. The doses and timing of injection were as follows: *Tbx3*^*CreERT2*^ *x Confetti* at postnatal day P1 (0.1 mg/ml)*, R26*^*CreERT2*^ *x Confetti* at P1 (0.067 mg/ml), *VE-Cadherin*^*CreERT2*^ *x Confetti* at P1 (0.067 mg/ml), *VE-Cadherin*^*CreERT2*^ *x Confetti* at P7 (0.1 mg/ml), WT1^CreERT2^;mTmG and WT1^CreERT2^;Wntless^flox/flox^ at P1, P2, P3 (2 mg/ml each), *Apelin*^*CreERT2*^ *x Confetti* at P1 (2 mg/ml), *Efnb2*^*CreERT2*^ *x Confetti* at P1 (2 mg/ml), *Apelin*^*CreERT2*^ *x mTmG* at P1, P2, P3 (2 mg/ml each), *Apelin*^*CreERT2*^ *x mTmG* at P6, P7 (2 mg/ml each), *Efnb2*^*CreERT2*^ *x mTmG* at P1, P2, P3 (2 mg/ml each), *Efnb2*^*CreERT2*^ *x mTmG* at P6, P7 (2 mg/ml each), *Lyve1*^*CreERT2*^ *x mTmG* at P1, P2, P3 (2 mg/ml each), *APJ*^*CreERT2*^ *x mTmG* at P1, P2, P3 (2 mg/ml each), *Esm1*^*CreERT2*^ *x mTmG* at P7 (2 mg/ml each), and *APJ*^*CreERT2*^ *x NICD at* P1, P2, P3 (2 mg/ml each). Tamoxifen was dissolved in sterile corn oil with 10% ethanol. One week following Tamoxifen induction, tissues were collected for initial labeling analysis based on previous studies^63^.

### EdU incorporation studies

C56BL/6 wildtype pups were intraperitoneally injected with EdU at P10 or P15 (1 mg EdU / 25 g body weight). 4 h later, pups were sacrificed and livers were collected, fixed in 4% paraformaldehyde overnight at 4 °C, and processed for vibrating microtome sectioning.

### Vibrating microtome sectioning and immunostaining

Thick section processing and immunostaining protocol was adapted from Snippert et al, 2011^64^. For embedding and sectioning, Precisionary compresstome equipment and tools were used (VF-310-0Z). Briefly, livers were isolated and fixed in 4% paraformaldehyde overnight at 4 °C followed by PBS washes (3 × 10 min). For sectioning adult samples and postnatal samples older than one week, the whole left median lobe or half of the left liver lobe was embedded curvature down. The embedding was performed in the manufacturer’s mold in 4% ultrapure low-melting agarose prepared in PBS. For the sectioning of livers from younger pups, the whole left median lobe was embedded floating over a microcentrifuge tube cap in the mold to obtain proper agarose levels for reliable sectioning. The samples were left in the embedding mold for 5 min followed by incubation in the provided ice block for 10 min. The adult samples and postnatal samples older than one week of age were sectioned at 100 µm, while samples from younger pups were sectioned at 50 µm. All sectioning was carried out at a speed set to 3 and a vibration set to 4.

For immunostaining, the compresstome sections were permeabilized in PBS containing Triton X-100 0.1% for 1 h, blocked in PBS with Triton X-100 0.1% containing normal donkey serum at 5% for 1 h, and incubated in primary antibodies listed in the antibodies section for 2 overnights at 4 °C. The sections were then washed in PBS containing Triton X-100 0.1% (4 x 15 min) and incubated in secondary antibodies raised in donkey conjugated to Alexa Fluor 488, 555, or 647 (1:400; Jackson Immunoresearch Labs) overnight at 4 °C. Next, the sections were washed in PBS containing Triton X-100 0.1% (4 x 15 min), incubated in DAPI solution in PBS (1:10000, ThermoFisher, D1306) for 30 min, and washed again for 15 min. Lastly, using a paintbrush, the sections were mounted on a slide with Prolong Gold with DAPI (Invitrogen) and protected with a coverslip.

For EdU incorporation studies, compresstome sections were treated with antigen retrieval solution following permeabilization and prior to blocking. Antigen retrieval was performed in Tris Buffer (Vector Labs H-3301) in a pressure cooker at low setting for 10 min. Following cool-down, sections were blocked. Next, EdU Click-it staining was performed according to the kit instructions (ThermoFisher C10337) for 1 h at room temperature. After this step, the sections were incubated in primary antibodies, and the regular compresstome staining protocol was followed.

### Paraffin sectioning and immunostaining

Livers were collected and fixed in 10% neutral buffered formalin overnight at room temperature. After three PBS washes, livers were dehydrated through 30 min incubations in a series of increasing Ethanol concentrations (30, 50, 70, 95, 100% x3) followed by HistoClear (x2) (National Diagnostics, HS-200) and a paraffin rinse. Livers were left in paraffin overnight at 65 °C. Next day, lobes were embedded in paraffin laying on the larger surface for coronal sectioning. Left median liver lobes were used. 5 µm paraffin sections were generated on the microtome. For in situ hybridization, water contact of sections did not exceed 1 min. Sections were dried at 40 °C on a slide warmer for up to 4 h. Paraffin immunostaining was performed as described before^15^. Briefly, deparaffinization was performed through 5 min incubations in HistoClear (x2), and decreasing concentrations of Ethanol (100% x2, 95, 70, 50%). After PBS rinse, antigen retrieval was performed in Tris Buffer (Vector Labs H-3301) in a pressure cooker at high setting for 30 min. Following cool-down, sections were blocked in a humidified chamber for 1 h in 5% normal donkey serum in PBS containing Triton X-100 0.1%, and incubated in primary antibodies in the same base solution overnight at 4 °C. Following three PBS washes, sections were incubated in secondary antibodies for 2 h at room temperature, washed in PBS three times, incubated in DAPI solution in PBS (1:10000, ThermoFisher, D1306) for 10 min, washed in PBS and mounted using Prolong Gold (with DAPI).

### Confocal imaging

Immunostained compresstome or paraffin liver sections were imaged using a Leica SP8 white light laser confocal. 10X air and 20X multi-immersion (with glycerol) objectives were used. Images of full liver sections were tiled and stitched by automatic merge using the Navigator view on the LasX Software (3.5.5.19976). Z stacks of images were projected into a 2-dimensional image by applying the maximum intensity projection in FIJI (ImageJ).

### In situ hybridization

In situ hybridization on postnatal liver samples was performed using the high-sensitivity RNA amplification and detection technology RNAscope (Advanced Cell Diagnostics). The samples were treated according to the manufacturer’s instructions. Briefly, the samples were fixed in 10% neutral buffered formalin overnight at room temperature. The samples were paraffin-embedded, and the left median liver lobes were sectioned within three days prior to and baked the day before in situ hybridization. The RNAscope chromogenic 2.5 HD Duplex Detection Kit was used. The antigen retrieval step was performed for 12 min. The signal detection steps were performed for 30 min. In situ hybridization paraffin sections were imaged at 10X or 20X magnification using a Zeiss Imager Z.2, and images were processed and analyzed in FIJI (ImageJ). The used probes are as follows: Mm-Procr-Ch2 (410321-C2), Mm-Tbx3-Ch1 (438211), Mm-Glul-Ch1 (426231), Mm-Cyp2f2-Ch2 (451851-C2), Mm-Axin2-Ch2 (400331-C2), Mm-Wnt2b-Ch1 (405031), Mm-Wnt4-Ch1 (401101), Mm-Wnt5b-Ch1 (405051), Mm-Pecam-Ch2 (316721-C2), Mm-Wnt2-Ch1 (313601), Mm-Vegfa-Ch1 (405131), Mm-Vegfc-Ch1 (492701), Mm-Wnt9b-Ch1 (405091), Mm-WT1-Ch2 (432711-C2), Mm-Wntless-Ch1 (405011), and Mm-Apelin-Ch2 (415371-C2).

### Volumetric immunofluorescence and light sheet imaging

Whole left median lobes were immunostained using a protocol adapted from the previously published iDisco protocol without Methanol pre-treatment^14^. The livers were perfused with fixative at the time of collection for thorough fixation. The perfusion was carried out by inserting a catheter connected to a perfusion pump through the inferior vena cava. Each liver was perfused with 10 ml PBS followed by 6 ml 4% paraformaldehyde. The samples were then fixed further in 4% paraformaldehyde overnight at 4 °C and washed in PBS (3 × 10 min). For iDisco pre-treatment, the samples were washed in PBS containing 0.2% TritonX-100, referred to as PTx.2 (1 h x 2), incubated in PTx.2 with 20% dimethyl sulfoxide overnight at 37 °C and then in PBS with 20% dimethyl sulfoxide containing a combination of detergents (0.1% Tween, 0.1% TritonX-100, 0.1% Deoxycholate, 0.1% NP40) overnight at 37 °C. The samples were washed in PTx.2 (1 h x 2) to complete the pre-treatment. For immunolabeling, the samples were incubated in permeabilization solution (PTx.2 containing 20% dimethyl sulfoxide and 2.3% glycine) for 2 days at 37 °C, in blocking solution (PTx.2 containing 10% dimethyl sulfoxide and 6% normal donkey serum) for 2 days at 37 °C, and in primary antibodies for a week at 37 °C. The primary antibodies were prepared in PBS containing 0.2% Tween20 and 10 µg/ml, referred to as PtwH, with 5% dimethyl sulfoxide and 3% normal donkey serum. The samples were then washed in PtwH (5 x 1 h) and left washing overnight. Next, the samples were incubated in secondary antibodies (prepared in PtwH containing 3% normal donkey serum) for a week at 37 °C followed by washes (5 x 1 h) and an overnight incubation in PtwH. The secondary antibodies used were raised in donkey and conjugated to Alexa Fluor 488, 555, or 647 (1:400; Jackson Immunoresearch Labs). These antibodies were centrifuged prior to diluting to avoid aggregates. For clearing, the samples were dehydrated in a methanol series with increasing concentration for 1 h each (20%, 40%, 60%, 80%, 100% x 2). The samples were left in 100% methanol overnight, and then incubated in 2:1 dichloromethane: methanol solution for 3 h. Finally, the samples were incubated in 100% dichloromethane for 15 min twice to rinse the methanol and transferred into dibenzyl ether in a completely filled tube. Except for this last step, all incubations were carried out in 4.5 ml solution volume. 5 ml Eppendorf tubes were used. For livers from 1-week-old pups, the samples were embedded in 1% agarose prepared in TAE buffer prior to clearing.

Whole liver lobe samples were imaged on a Miltenyi/LaVision Biotec Ultramicroscope II. Volumetric images were generated using the Imaris (Bitplane) software, version 10.0.0. Deconvolution was performed for improved visualization of volumetric images.

### Antibodies

The following primary antibodies were used at indicated concentrations for the immunostaining of compresstome sections, iDisco whole lobe samples, or paraffin sections: goat anti-EPCR (R&D Systems, AF2749, 1:100), rat anti-Endomucin (Santa Cruz Biotech, sc-65495, 1:100), rabbit anti-phospho histone H3 (Milipore Sigma, 06-570, 1:100), mouse anti-Hepatocyte nuclear factor 4 alpha (Abcam, ab41898, 1:200), chicken anti-GFP (Abcam, ab13970, 1:500), rabbit anti-RFP (Rockland, 600-401–379, 1:200), rabbit anti-Sox9 (Millipore, AB5535, 1:100), rabbit anti-Lyve1 (Novus Biologicals, NB600-1008, 1:500), and rabbit anti-ERG (Abcam, ab110639, 1:200).

### Image processing, analysis, and quantification

Tiled and stitched full-section confocal images of z-stacks (30–50 um) were converted into maximum projection images, and the projected images were used for further analyses unless otherwise noted below. Whole left median lobes were used in all experiments unless otherwise noted below or in the figure legends. Same thickness images were used across samples for quantification of cell numbers or the comparison of fluorescence intensity. Images used for comparison were acquired with the same imaging settings. For DAPI normalization, the DAPI-stained area was measured following intensity thresholding. For the presentation of EPCR and GFP immunostaining in figures, the images were despeckled in FIJI (ImageJ). On full liver section images in Figs. 1c, 2i, l, and Suppl. Fig. 4b, the autofluorescence signal due to trapped air bubbles outside of the tissue section was masked during figure assembly for presentation purposes. Image quantifications are described in detail below. Unless individual values were plotted as denoted in the figure legend, the average value derived from 2–3 sections of each sample was calculated and presented as a data point.

#### Lobule size quantification

EPCR-immunostained full liver section images were cropped into a single lobule size for all lobules identified. 140–210 lobules from a total of 3 sections per sample and from 3 mice at each age were analyzed in Fig. 1. In Wntless^meso^ and NICD^venous^ litters, lobules were segregated based on location at the periphery or in the core. The images from each group were run through a custom MatLab script generated in this study to assess the number of nuclei and absolute area per lobule.

#### Quantification of lobule numbers

Volumetric iDisco samples immunostained for EPCR were used for the quantification of mature lobules in the left median lobe. The lobules were manually counted on FIJI (ImageJ). The mature lobule was defined as one with boundaries marked by EPCR immunostaining and a central vein lumen, which can be identified against the background tissue signal. It was empirically determined that lobule numbers quantified on sections accurately represent the total number of lobules when two samples are being compared, and thus the comparison of lobule numbers in Wntless^meso^ and NICD^venous^ litters was performed on sections. 2–3 sections from 4 Wntless^meso^ and 4 control littermates were analyzed. 2 sections from 5 NICD^venous^ mice and 7 control littermates were analyzed.

#### Edge measurements at neonatal ages

On EPCR-immunostained full liver section images, the EPCR-positive portal vessel structures closest to the edge of the section were manually traced and connected. This tracing partitioned a thin layer of tissue that constitutes the edge and the rest of the tissue that constitutes the core.

#### Periphery measurements at adulthood

On EPCR-immunostained full liver section images, the lobule boundaries were manually traced at one lobule distance along the outer edge of the liver lobe, counting three lobules up from the corner of the distal tip. In cases where the lobule hexagon is not complete at the section edge, it was counted as a lobule when the corresponding central vein is discernable.

#### Quantification of endothelial EdU incorporation

On EdU and ERG immunostained full liver sections, the two channels were separated in FIJI (ImageJ). This was followed by intensity thresholding and mask generation for each channel. The EdU and ERG masks were then combined via the AND function to identify the pixels positive for both EdU and ERG. Next, Analyze Particles function was applied at 6–25 size and 0.5–1.0 circularity thresholds to identify the nuclei positive for both, and separately to the ERG mask to identify all endothelial cell nuclei. The ratio of double positive nuclei to ERG positive nuclei was calculated and presented as percentage.

#### Quantification of mitosis

Phospho-histone H3 immunostained full liver section images were used. In FIJI (ImageJ), intensity thresholding and size filtering were applied to filter out noise. The same thresholds were used across samples under comparison. The “analyze particles” function of FIJI (ImageJ) was applied to the post-filtering images to identify the nuclei with pHH3 staining. The obtained numbers were normalized to the tissue area defined as the DAPI-stained area. The normalized numbers represent the mitotic or pHH3 index. Relative numbers were generated for plots by multiplying the DAPI-normalized ratio by 10^5^ or 10^6^ as denoted. To quantify the distribution of mitosis, a similar approach was used, accompanied by periphery identification. The corresponding EPCR-stained split image was used to identify the periphery as described above. The corresponding particle analysis image and the DAPI split image were both partitioned into the periphery and the complementary core in sync with the corresponding EPCR image. The number of particles was normalized to the corresponding DAPI-stained area for the periphery and the core separately, and the DAPI-normalized ratios were multiplied by 10^5^ or 10^6^, as denoted, to generate relative numbers for plots.

#### Quantification of hepatocyte mitosis

Phospho-histone H3 (pHH3) and hepatocyte nuclear factor 4-alpha (Hnf4a) immunostained full liver sections were used. The two channels were separated in FIJI (ImageJ), followed by intensity thresholding and size filtering to filter out noise. Next, masks were generated for each channel. For the pHH3 mask, Dilate, Close, and Erode functions were applied in this order to account for a spotty yet true signal. The final pHH3 mask and the Hnf4a mask were combined via the AND function to identify the pixels positive for both signals. Analyze Particles was applied to the combined image to identify the cells positive for both pHH3 and Hnf4a, and separately to the Hnf4a mask. The ratio of double-positive cells to Hnf4a-positive cells was calculated and presented as a percentage.

#### Hepatocyte clone size quantification

Hepatocyte clone size was assessed by manual cell counting on slices of z-stacks of RFP- and GFP-immunostained Tbx3^CreERT2^; Confetti or R26 ^CreERT2^; Confetti samples in FIJI (ImageJ). For Tbx3^CreERT2^; Confetti, 126 clones in the core and 41 clones in the periphery from a total of 3 sections collected from 3 mice were analyzed. For R26 ^CreERT2^; Confetti, 735 clones in the core and 928 clones in the periphery from a total of 3 sections collected from 3 mice were analyzed. Cells of the same color (RFP or GFP) and with the same reporter localization (nuclear, cytoplasmic, or membrane) within the empirically determined one-cell-distance were considered belonging to the same clone. Single cells were excluded from the analysis.

#### Endothelial clone size quantification

Endothelial clone size was assessed on maximum projection images of RFP-immunostained VE-Cadherin^CreERT2^; Confetti samples. 457 (labeled at 1 day old) and 702 (labeled at 1 week old) clones from a total of 2–3 sections per sample and from 3 mice at each age were analyzed. Single cells were excluded from the analysis. The area of each clone was measured using a MATLAB script for endothelial clone area quantification generated in this study. Rarely, clones were erroneously called by the script due to high background in images and discarded post-data collection (0.6% error rate). The clones were then binned by area measurement, and the number of clones in each bin were plotted to represent clone size distribution. The irregular right tail of the histogram was used to identify rare, large clones, which were then defined as giant clones. For comparison of clone size between the core and the periphery, the same script was used in combination with EPCR-guided periphery identification. Out of 457 clones, 128 localized to the periphery, while 329 localized to the core. The periphery was manually traced using the corresponding EPCR-stained split images as part of the MATLAB script.

#### Labeling efficiency quantification

The labeling efficiency was measured by manually counting the number of labeling events on maximum projection images of RFP- and GFP-immunostained VE-Cadherin^CreERT2^; Confetti samples in FIJI (ImageJ). The number of labeling events was defined as the sum of the number of single cells and the number of clones, where each clone is counted as one labeling event. This number was normalized to the tissue area defined as the DAPI-stained area. Relative numbers were generated for plots by multiplying the DAPI-normalized ratio by 10^6^.

#### Quantification of central vein-derived clone size

The size of central-vein derived clones was assessed on maximum projection images of RFP- and GFP-immunostained Apelin^CreERT2^; Confetti samples. 70 clones in the core and 30 clones in the periphery from a total of 3 sections per sample and from 3 mice were analyzed. The cells were manually counted in FIJI (ImageJ). Single cells were excluded from the analysis (158 single cells out of 258 total labeling events).

#### Quantification of central vein-derived expansion

The extent of central vein-derived expansion was measured using intensity thresholding on maximum projection images of GFP-immunostained Apelin^CreERT2^; mTmG samples in FIJI (ImageJ). 1–3 sections from 3 mice at each age were analyzed. The GFP-stained area was normalized to the tissue area defined as the DAPI-stained area and plotted as a percentage. The number of veins with different central-vein derived expansion outcomes was manually evaluated on maximum projection images of GFP-immunostained Apelin^CreERT2^; mTmG samples in FIJI (ImageJ).

#### Quantification of portal vessel-derived clone size

The size of portal-vessel derived clones was assessed on maximum projection images of RFP- and GFP-immunostained Efnb2^CreERT2^; Confetti samples. Despite maximum Tamoxifen dosage, the labeling events in these mice remained very low. Assessment of 3 sections per sample from 3 mice yielded only 17 clones in the core and 8 clones in the periphery. The number of cells in these clones was manually assessed in FIJI (ImageJ). Single cells were excluded from the analysis (29 single cells out of 54 total labeling events).

#### Quantification of central vein labeling outcomes

The outcomes of central vein labeling were assessed manually on maximum projection images of RFP- and GFP-immunostained Apelin^CreERT2^; Confetti samples in FIJI (ImageJ). A total of 77 clones and 59 single cells from 1–3 sections per sample collected from 4 mice were analyzed.

#### Quantification of portal vessel-derived expansion

The extent of portal vessel-derived expansion was measured using intensity thresholding on maximum projection images of GFP-immunostained Efnb2^CreERT2^; mTmG samples in FIJI (ImageJ). 2 sections from 2 mice at each age were analyzed. The GFP-stained area was normalized to the tissue area defined as the DAPI-stained area and plotted as a percentage.

#### Quantification of the extent of vein-derived expansion in NICD^venous^ mice

The extent of vein-derived expansion was measured using intensity thresholding on maximum projection images of GFP-immunostained NICD^venous^; mTmG and littermate samples in FIJI (ImageJ). 3 sections from 2 males and 2 females were analyzed, each for NICD^venous^ and control mice. The GFP-stained area was normalized to the tissue area defined as the DAPI-stained area and plotted as a percentage.

### Statistical analyses

Statistical analyses were performed using Prism 9 (Graphpad Software 9.5.1). Unpaired *t*-test was applied to comparisons between two conditions with similar sample sizes except that of average sample readouts between the core and the periphery. The latter were analyzed using the ratio paired *t*-test. When comparing two conditions with different sample sizes (difference > 50%), Welch’s *t*-test was performed to account for different variances between datasets. Ordinary one-way Anova analysis and Tukey’s multiple comparisons test was applied to comparisons across more than two conditions unless variances differed. Brown-Forsythe Anova analysis and Dunnett’s multiple comparisons test were applied to comparisons across more than two datasets with differing variances. Liver size parameters were tested for litter effect based on a previously published method^65^ and, when litter effect was present, the statistical test result from two-way Anova replaced the one from the unpaired *t*-test. All tests were two-sided. *P* < 0.05 was considered statistically significant. The significance annotations are *p-value < 0.05, **p-value < 0.01, ***p-value < 0.001, ****p-value < 0.0001. Sample size was not predetermined with statistical methods. Previous studies using similar experimental approaches guided the decisions on sample size. The investigators were blinded during the treatment, collection, immunostaining, imaging, and quantification of Wntless^meso^ and NICD^venous^ studies, and during the quantification of the number of lobules in wild types. The rest of the experiments were performed without blinding.

### Reporting summary

Further information on research design is available in the Nature Portfolio Reporting Summary linked to this article.

## Supplementary information

Supplementary Information

Peer Review file

Description of Additional Supplementary Files

Supplementary Movie 1

Supplementary Movie 2

Supplementary Movie 3

Reporting Summary

## Source data

Source data

## Data Availability

Source data are provided with this paper.

## Code availability

The custom Matlab script to quantify endothelial clone size and distribution has been deposited to Github and can be accessed at https://github.com/berfinazizoglu/Liver_Endothelial_Clones (10.5281/zenodo.17095107)^66^.
