# Supplementary material for: Vascularization of neonatal liver lobules presages adult liver size
Source: Nat Commun. 2025 Nov 13;16:9989. doi: 10.1038/s41467-025-64930-w (PMC12615705; doi:10.1038/s41467-025-64930-w)
Supplement: Supplementary file 1 — Supplementary Information [file 41467_2025_64930_MOESM1_ESM.pdf]

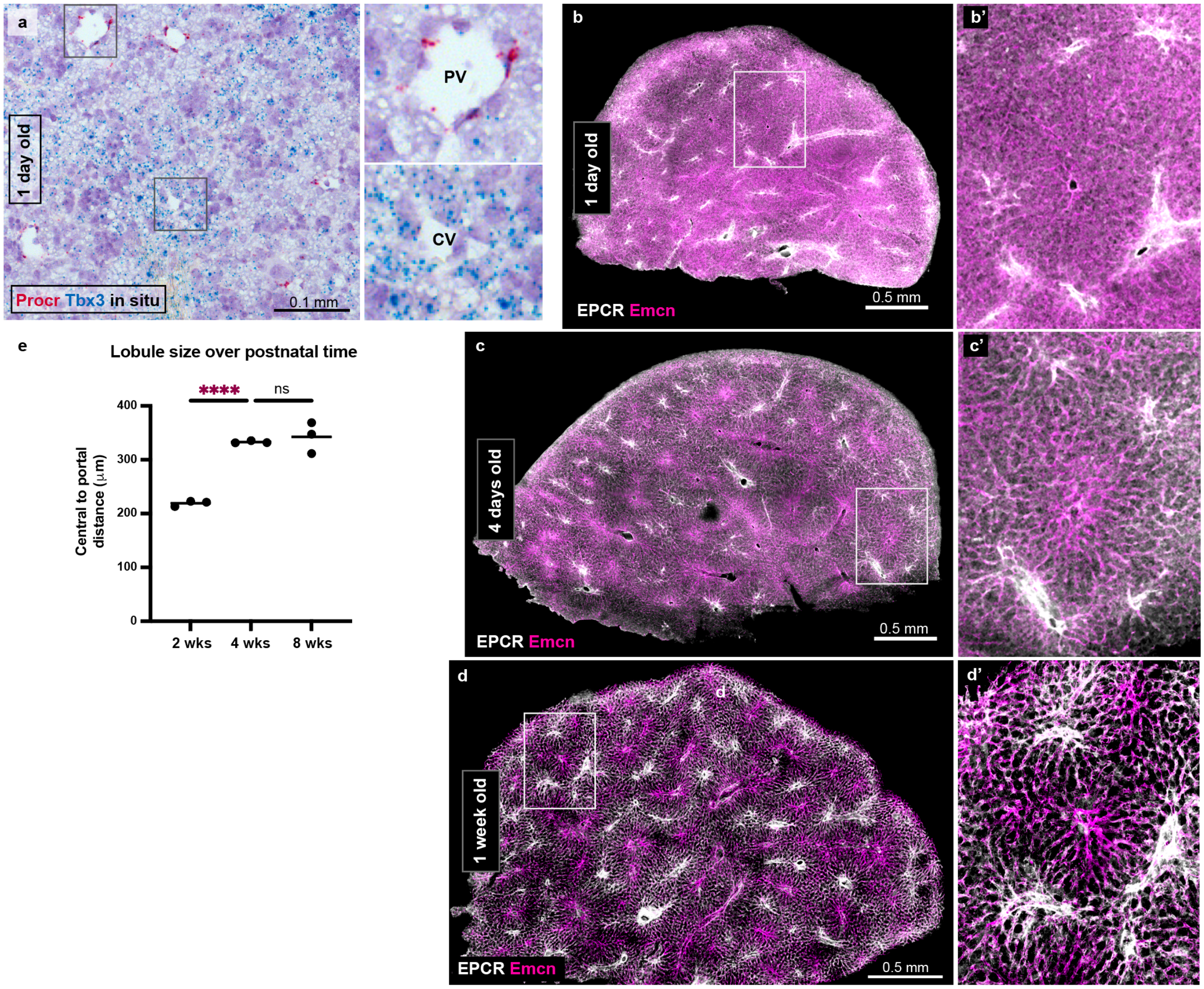

Extended Data Fig. 1 Temporal assessment of postnatal liver lobules. a, *In situ* hybridization on a representative wildtype liver section at 1 day old for the lobule boundary marker EPCR and *T-box transcription factor 3 (Tbx3)* expressed in zone 2 and 3 hepatocytes. **b-d',** Thick liver sections immunostained for the lobule boundary marker EPCR and the venous marker Endomucin in 1-day-old, 4-day-old, and 1-week-old wildtype mice. **e,** Plot shows individual data points for Fig. 1f on the distance from the central vein to portal vessels in livers of 2 week-, 4 week-, and 8-week-old male mice. Black bars represent the mean. A total of n=210 (2 weeks), n=147 (4 weeks), and n=162 (8 weeks) lobules were scored in 3 mice per group. Brown-Forsythe Anova test and Dunnett's multiple comparisons test, adjusted p-values from left to right: < *0.0001, 0.8247*. PV, portal vessel. CV, central vein. Source data are provided in the Supp. Figures 1-3 Source Data file.

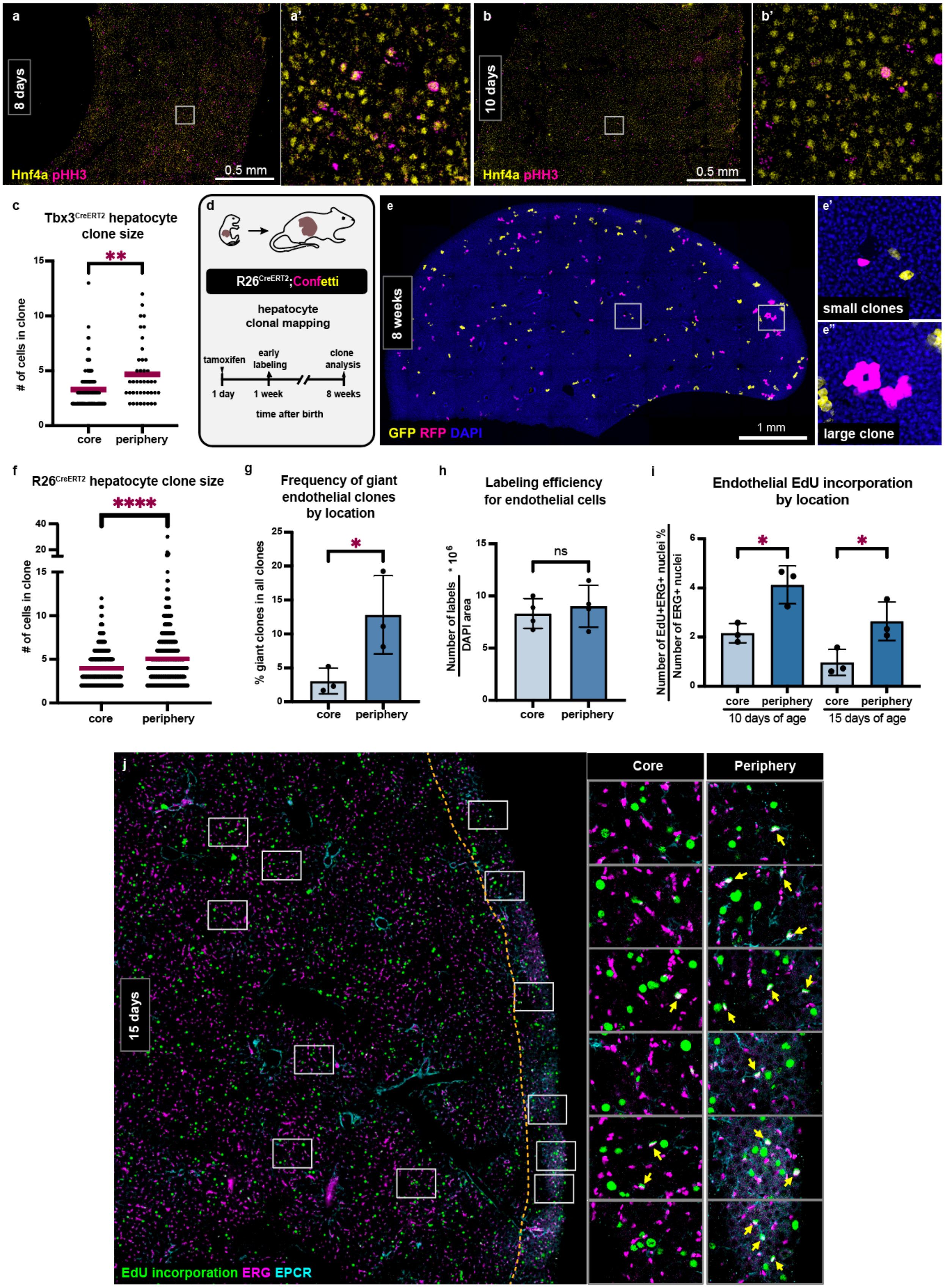

a
8 days
Hnf4a pHH3
0.5 mm
a'
b
10 days
Hnf4a pHH3
0.5 mm
b'
c
Tbx3CreERT2 hepatocyte clone size
# of cells in clone
core
periphery
d
R26CreERT2;Confetti
hepatocyte clonal mapping
tamoxifen
early labeling
clone analysis
1 day
1 week
8 weeks
time after birth
e
8 weeks
GFP RFP DAPI
1 mm
e'
small clones
e''
large clone
f
R26CreERT2 hepatocyte clone size
# of cells in clone
core
periphery
g
Frequency of giant endothelial clones by location
% giant clones in all clones
core
periphery
h
Labeling efficiency for endothelial cells
Number of labels / DAPI area * 10^6
ns
core
periphery
i
Endothelial EdU incorporation by location
Number of EdU+ERG+ nuclei % Number of ERG+ nuclei
core
periphery
10 days of age
core
periphery
15 days of age
j
15 days
EdU incorporation ERG EPCR
Core
Periphery

15

Extended Data Fig. 2 Assessment of hepatocye and endothelial expansion in the postnatal liver. a-b', Paraffin sections of 8-day-old and 10-day-old male mice immunostained for Hepatocyte nuclear factor 4a (Hnf4a) and phospho-histone H3 (pHH3) to analyze hepatocyte proliferation. **c,** Quantification of hepatocyte clone size measured as the number of cells per clone in the periphery compared to the core in 8-week-old Tbx3^{CreERT2};Confetti livers. n=126 (core) and n=41 (periphery) clones from 3 mice. Red bars represent the mean values. Welch's t test, *p-value = 0.0035*. **d,** Schematic summary of the experimental design to map hepatocyte expansion using R26^{CreERT2};Confetti. **e-e'',** R26^{CreERT2};Confetti thick liver section immunostained for GFP and RFP to visualize the clones at 8 weeks. **f,** Quantification of hepatocyte clone size assessed by the number of cells per clone in the periphery compared to the core in 8-week-old R26^{CreERT2};Confetti livers. n=735 (core) and n=928 (periphery) clones from 3 mice. Red bars represent the mean values. Welch's t test, *p-value < 0.0001*. **g,** Quantification of the frequency of giant clones (area > 30,000 μm^2) in the periphery compared to the core. Unpaired t test, *p-value = 0.0493*. **h,** Quantification of endothelial cell labeling efficiency measured as the number of labeling events normalized to the tissue area in 1-week-old VE-Cadherin^{CreERT2};Confetti livers. n=4 mice per group, mean values ± s.d. Unpaired t test, *p-value = 0.5933*. **i,** Quantification of endothelial EdU incorporation in the periphery compared to the core as a result of 4 hour pulse-chase at 10 and 15 days of age. n=3 mice per group, mean values ± s.d. One-way Anova, *adjusted p-value at P10 = 0.0110; adjusted p-value at P15 = 0.0247*. **j,** Thick liver section from a 15-day-old mouse immunostained for EdU incorporation, ERG to mark endothelial nuclei, and EPCR to mark lobule boundaries. The dotted line delineates the periphery. Areas denoted by boxes are magnified to the right, categorized into the periphery or the core. Yellow arrows show ERG+EdU+ nuclei of replicating endothelial cells. ns, not significant. Source data are provided in the Supp. Figures 1-3 Source Data file.

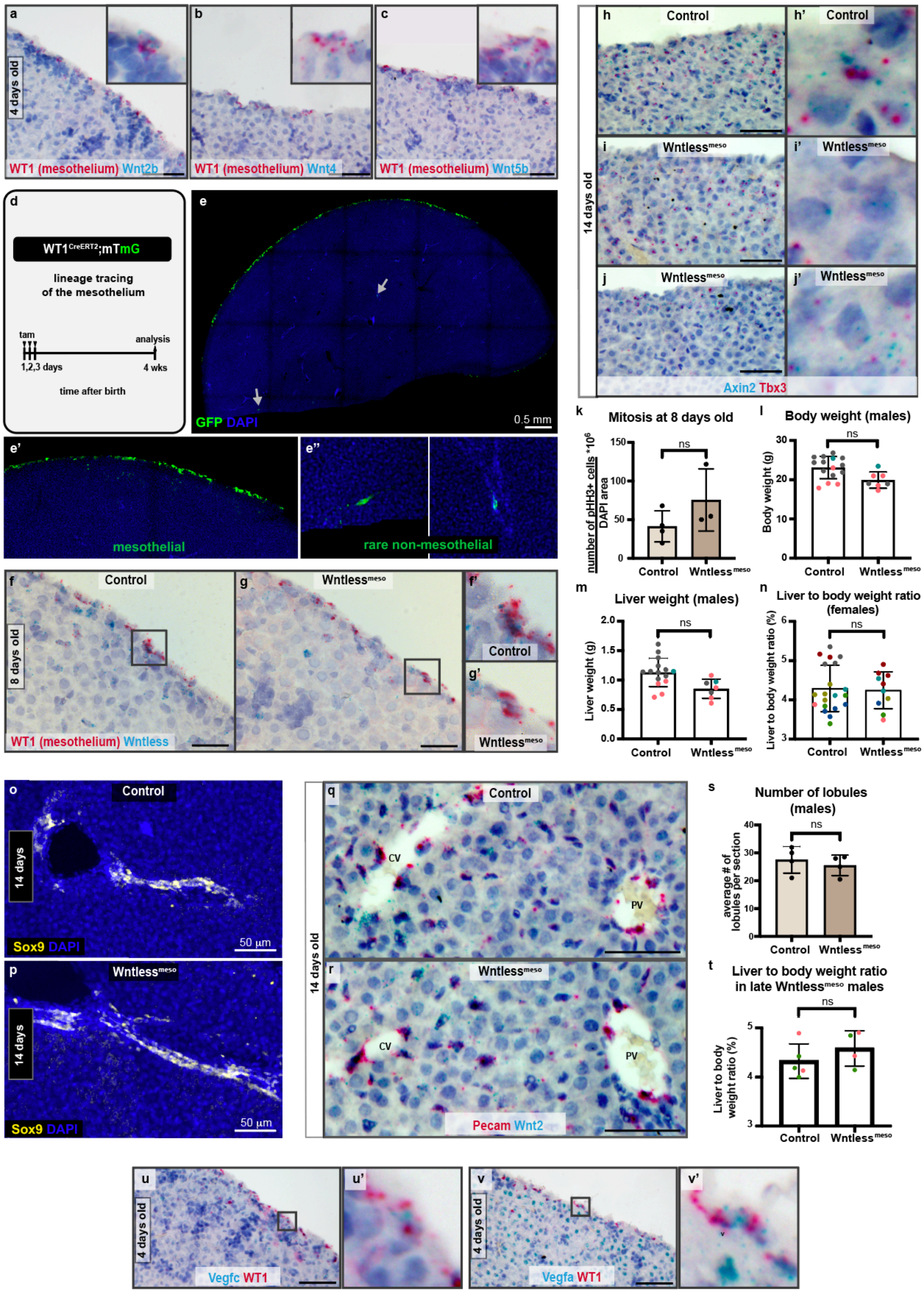

4 days old
WT1 (mesothelium) Wnt2b
WT1 (mesothelium) Wnt4
WT1 (mesothelium) Wnt5b
WT1CreERT2;mTmG
lineage tracing of the mesothelium
tam
analysis
1,2,3 days
4 wks
time after birth
GFP DAPI
0.5 mm
mesothelial
rare non-mesothelial
Control
Wntlessmeso
8 days old
WT1 (mesothelium) Wntless
14 days old
Axin2 Tbx3
Mitosis at 8 days old
number of pHH3+ cells *10^6 DAPI area
Body weight (males)
Body weight (g)
Liver weight (males)
Liver weight (g)
Liver to body weight ratio (females)
Liver to body weight ratio (%)
ns
14 days
Sox9 DAPI
50 µm
CV
PV
Pecam Wnt2
Number of lobules (males)
average # of lobules per section
Liver to body weight ratio in late Wntlessmeso males
Liver to body weight ratio (%)
Vegfc WT1
Vegfa WT1

39

Extended Data Fig. 3 Characterization of mesothelial-specific Wntless loss of function in the postnatal liver. a-c, *In situ* hybridization on 4-day-old liver sections for Wnt2b, Wnt4, and Wnt5b along with mesothelial-specific *Wilms Tumor 1 (WT1)*. Scale bars, 50 µm. **d,** Schematic summary of the experimental design to assess cell type specificity in $WT1^{CreERT2}$ mice by lineage-tracing with $WT1^{CreERT2}$;mTmG. **e-e''** $WT1^{CreERT2}$;mTmG thick liver section immunostained for GFP to visualize lineage labeling at 4 weeks. Arrows in **e** point at rare non-mesothelial labeling. **f-g',** *In situ* hybridization on liver sections for *Wntless* and the mesothelial-specific *Wilms Tumor 1 (WT1)* from 8-day-old control and mesothelial-specific Wntless knock-out, or $Wntless^{meso}$, mice. Scale bars, 25 µm. **h-j',** *In situ* hybridization on liver sections from 14-day-old control and $Wntless^{meso}$ mice for Wnt target genes Axin2 and Tbx3. The periphery of the liver is shown. Scale bars, 50 µm. **k,** Quantification of overall mitotic index in 8-day-old control (n=4) and $Wntless^{meso}$ (n=3) mice, mean values ± s.d. Unpaired t test, *p-value = 0.1954*. **l-m,** Body weight and liver weight measured in 8-week-old male control (n=15) and $Wntless^{meso}$ (n=7) mice. Mice are color-coded by litter. Mean values ± s.d. Two-way Anova and Tukey's multiple comparisons test performed. Adjusted *p-value for body weight = 0.5929, for liver weight = 0.5118*. **n,** Liver-to-body weight ratio of 8-week-old female control (n=20) and $Wntless^{meso}$ (n=10) mice. Mice are color-coded by litter. Mean values ± s.d. Two-way Anova and Tukey's multiple comparisons test performed. Adjusted *p-value = 0.1766*. **o-p,** Thick liver sections of 14-day-old control and $Wntless^{meso}$ male mice immunostained for the biliary marker Sox9. **q-r,** *In situ* hybridization on 14-day-old liver sections of control and $Wntless^{meso}$ male mice probed for endothelial Pecam and Wnt2. Scale bars, 50 µm. **s,** Quantification of the number of lobules in 8-week-old control (n=2) and $Wntless^{meso}$ (n=4) mice, mean values ± s.d. Unpaired t test, *p-value = 0.8545*. **t,** Liver-to-body weight ratio of 8-week-old control (n=5) and $Wntless^{meso}$ (n=4) males. Mice are color-coded by litter. Mean values ± s.d. Unpaired t test, *p-value = 0.3204*. **u-v',** *In situ* hybridization on 4-day-old liver sections for Vegf-c and Vegf-a along with mesothelial-specific *Wilms Tumor 1 (WT1)*. Scale bars, 50 µm. ns, not significant. PV, portal vessel. CV, central vein. Source data are provided in the Supp. Figures 1-3 Source Data file.

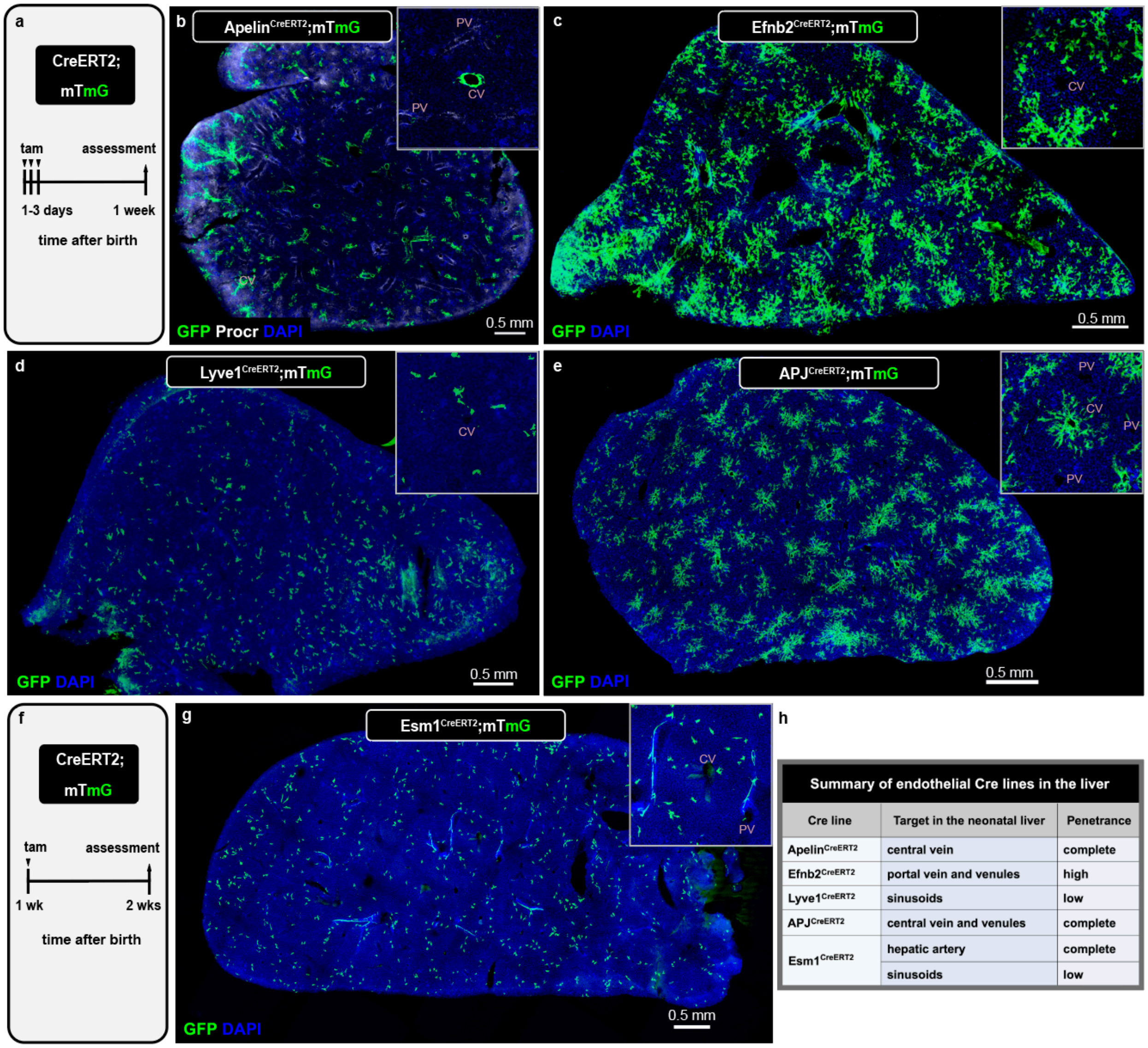

Summary of endothelial Cre lines in the liver		
Cre line	Target in the neonatal liver	Penetrance
Apelin^{CreERT2}	central vein	complete
Efnb2^{CreERT2}	portal vein and venules	high
Lyve1^{CreERT2}	sinusoids	low
APJ^{CreERT2}	central vein and venules	complete
Esm1^{CreERT2}	hepatic artery	complete
	sinusoids	low

Extended Data Fig. 4 Genetic targeting of vessel subtypes in the postnatal liver. a, Schematic summary of the experimental design to label different vessel subtypes as a whole compartment using CreERT2 lines in the postnatal liver. **b,** Apelin^{CreERT2};mTmG thick liver section immunostained for GFP and the lobule boundary marker EPCR at 1 week. **c-e,** Efnb2^{CreERT2};mTmG, Lyve1^{CreERT2};mTmG, and APJ^{CreERT2};mTmG thick liver sections immunostained for GFP at 1 week. **f,** Schematic summary of the experimental design to label vessels in Esm1^{CreERT2};mTmG mice. **g,** Esm1^{CreERT2};mTmG thick liver section immunostained for GFP at 2 weeks. **h,** Summary table of labeling outcomes for the vascular subtype Cre lines in the postnatal liver. DAPI in blue depicts nuclei. tam, tamoxifen. PV, portal vessel. CV, central vein.

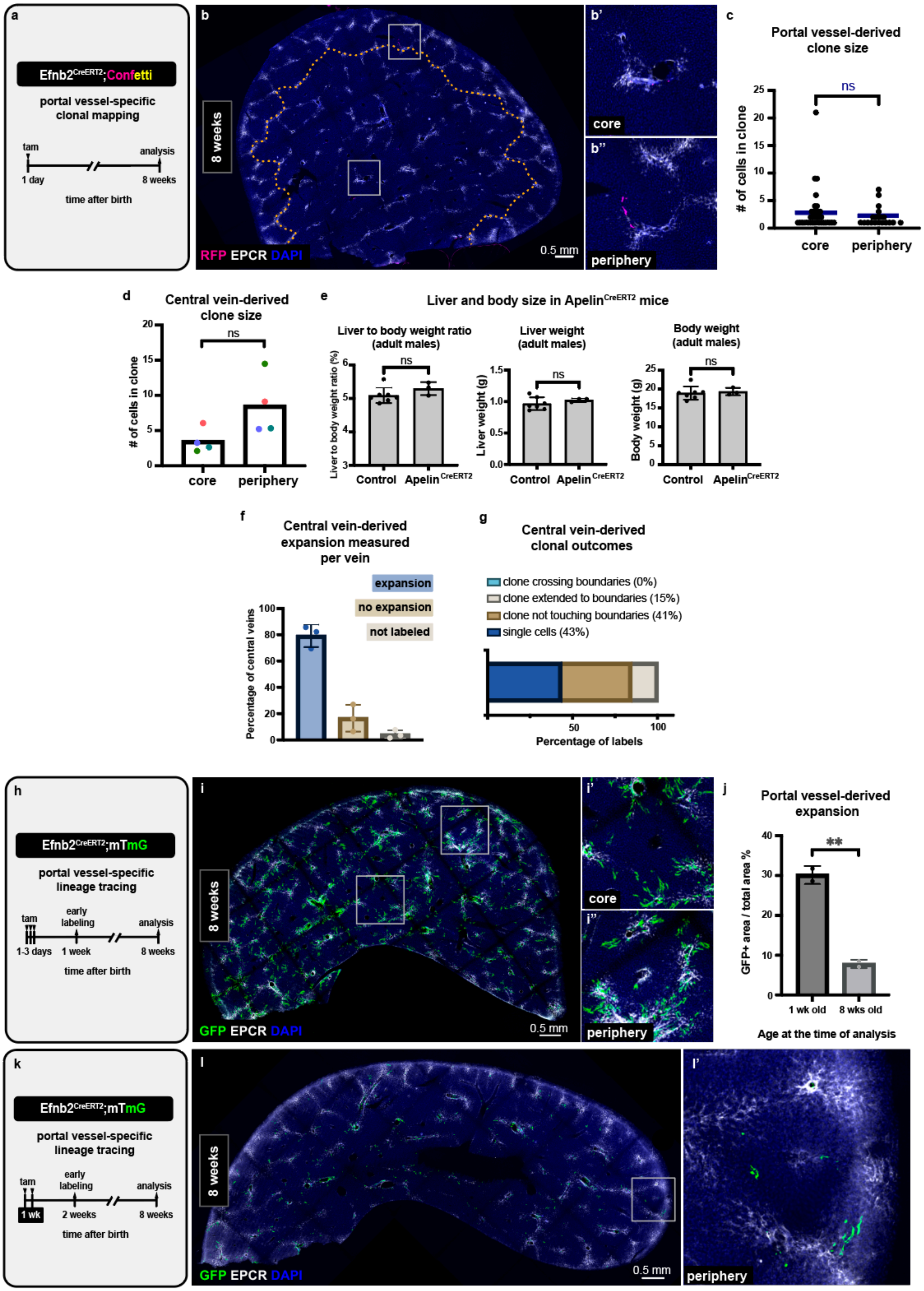
a
Efnb2CreERT2;Confetti
portal vessel-specific clonal mapping
tam
analysis
1 day
8 weeks
time after birth
b
8 weeks
RFP EPCR DAPI
0.5 mm
b'
core
b''
periphery
c
Portal vessel-derived clone size
ns
# of cells in clone
core
periphery
d
Central vein-derived clone size
ns
# of cells in clone
core
periphery
e
Liver and body size in ApelinCreERT2 mice
Liver to body weight ratio (adult males)
Liver to body weight ratio (%)
Liver weight (adult males)
Liver weight (g)
Body weight (adult males)
Body weight (g)
Control
ApelinCreERT2
f
Central vein-derived expansion measured per vein
expansion
no expansion
not labeled
Percentage of central veins
g
Central vein-derived clonal outcomes
clone crossing boundaries (0%)
clone extended to boundaries (15%)
clone not touching boundaries (41%)
single cells (43%)
Percentage of labels
h
Efnb2CreERT2;mTmG
portal vessel-specific lineage tracing
tam
early labeling
analysis
1-3 days
1 week
8 weeks
time after birth
i
8 weeks
GFP EPCR DAPI
0.5 mm
i'
core
i''
periphery
j
Portal vessel-derived expansion
GFP+ area / total area %
**
1 wk old
8 wks old
Age at the time of analysis
k
Efnb2CreERT2;mTmG
portal vessel-specific lineage tracing
tam
early labeling
analysis
1 wk
2 weeks
8 weeks
time after birth
l
8 weeks
GFP EPCR DAPI
0.5 mm
l'
periphery

84

Extended Data Fig. 5 Contribution of portal vessels and the central vein to nascent lobule vasculature. a, Schematic summary of the experimental design to map portal vessel-derived clonal expansion using Efnb2^{CreERT2};Confetti. **b-b'',** Efnb2^{CreERT2};Confetti thick liver section immunostained for RFP to mark the clones and EPCR to mark the lobule boundaries at 8 weeks following induction at postnatal day 1. The dotted line delineates the periphery. **c,** Quantification of portal vessel-derived clone size measured as the number of cells per clone in the periphery (n=8) compared to the core (n=17) from 3 Efnb2^{CreERT2};Confetti mice at 8 weeks. Blue bars represent mean values. Welch's t test, *p-value = 0.3828*. **d,** Central vein-derived clone size plotted as average values for individual mice, same dataset as in Fig. 4c. Unpaired t test, *p value = 0.0764.* **e,** Liver-to-body weight ratio, liver weight, and body weight assessed in 8-week-old Apelin^{CreERT2} males. Unpaired t test, *p values for liver-to-body weight ratio = 0.2200, for liver weight = 0.3686, for body weight = 0.7398.* **f,** Quantification of the percentage of central veins with different expansion or labeling behaviors (expansion at 79 %, no expansion at 17 %, and not labeled at 3 %) in 8-week-old Apelin^{CreERT2};mTmG mice. 637 central veins scored from n=3 mice . **g,** Summary graph of the percentage of central vein-derived clonal outcomes categorized by the spatial pattern of clones relative to lobule boundaries in 8-week-old Apelin^{CreERT2};Confetti mice. n=272 labels (clones and single cells) were scored from 4 mice. **h,** Schematic summary of the experimental design to lineage trace portal vessels induced at 1 day (injections at postnatal days 1-3) using Efnb2^{CreERT2};mTmG. **i-i'',** Efnb2^{CreERT2};mTmG thick liver section immunostained for GFP and the lobule boundary marker EPCR at 8 weeks following induction at 1 day. **j,** Quantification of portal vessel-derived expansion at 8 weeks compared to the early labeling at 1 week in Efnb2^{CreERT2};mTmG mice. n=2 mice per group, mean values ± s.d. Unpaired t test, *p-value = 0.0061*. **k,** Schematic summary of the experimental design to lineage trace portal vessels induced at 1 week (injections on postnatal days 6 and 7). **l-l',** Efnb2^{CreERT2};mTmG thick liver section immunostained for GFP and the lobule boundary marker EPCR at 8 weeks following induction at 1 week. DAPI in blue depicts nuclei. tam, tamoxifen. ns, not significant. Source data are provided in the Supp. Figures 5-7 Source Data file.

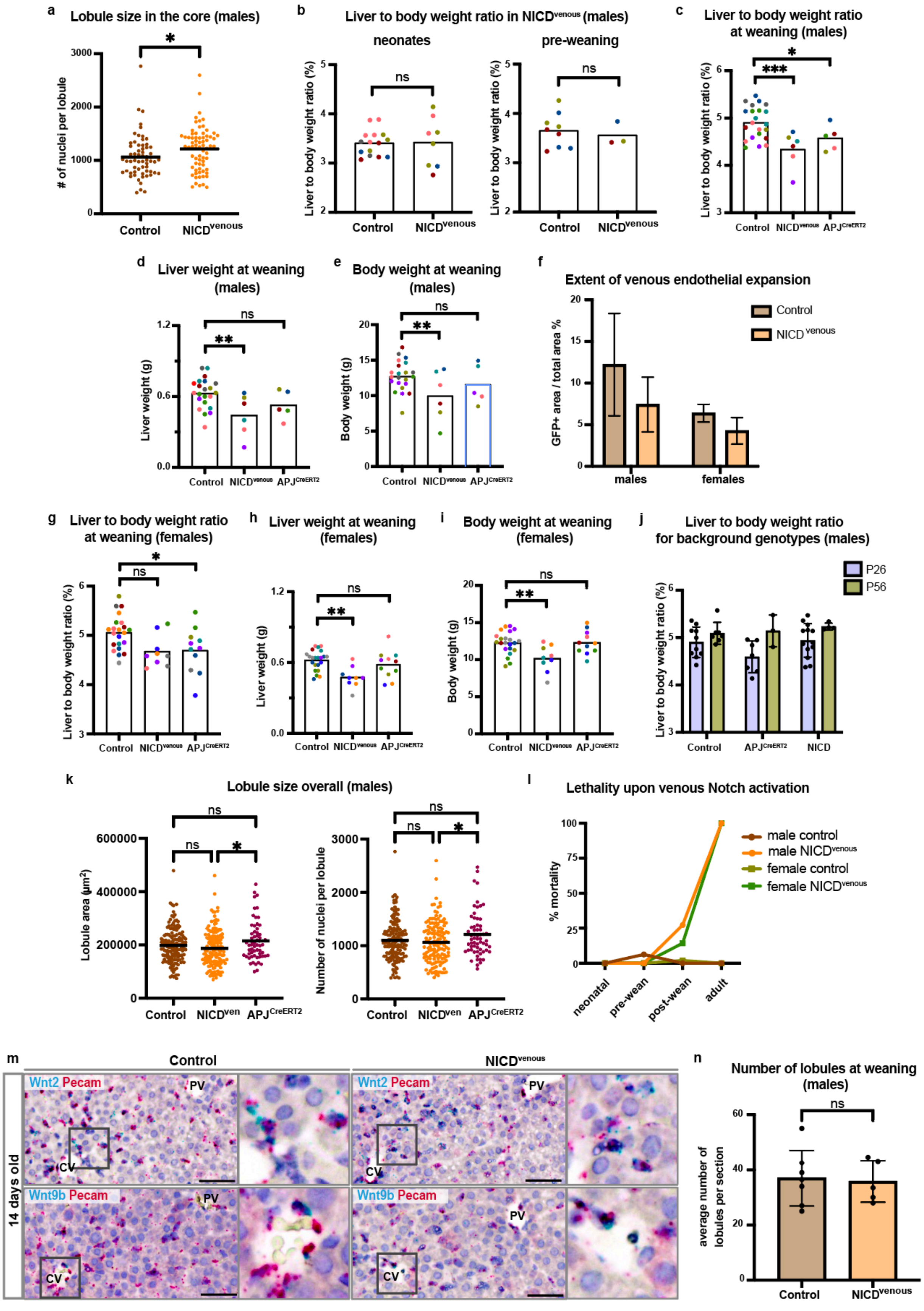
a
Lobule size in the core (males)
# of nuclei per lobule
Control
NICDvenous
b
Liver to body weight ratio in NICDvenous (males)
neonates
pre-weaning
Liver to body weight ratio (%)
ns
c
Liver to body weight ratio at weaning (males)
APJCreERT2
d
Liver weight at weaning (males)
Liver weight (g)
e
Body weight at weaning (males)
Body weight (g)
f
Extent of venous endothelial expansion
GFP+ area / total area %
males
females
g
Liver to body weight ratio at weaning (females)
h
Liver weight at weaning (females)
i
Body weight at weaning (females)
j
Liver to body weight ratio for background genotypes (males)
P26
P56
NICD
k
Lobule size overall (males)
Lobule area (µm²)
Number of nuclei per lobule
NICDven
l
Lethality upon venous Notch activation
% mortality
male control
male NICDvenous
female control
female NICDvenous
neonatal
pre-wean
post-wean
adult
m
14 days old
Wnt2 Pecam
Wnt9b Pecam
PV
CV
n
Number of lobules at weaning (males)
average number of lobules per section

Extended Data Fig. 6 Characterization of venous angiogenesis inhibition in the postnatal liver. a, Quantification of lobule size measured by number of nuclei per lobule in APJ^{CreERT2};mTmG;NICD (NICD^{venous}, n=5) and littermate control (n=5) males at 26 days in the core of the liver. Black bars represent mean values. Unpaired t test, *p-value = 0.0320*. **b,** Liver to body weight ratio measured in 8-day-old control (n=15) and NICD^{venous} (n=8) male neonates, and in 14-day-old control (n=9) and NICD^{venous} (n=3) male pre-weaning pups. Mice are color-coded by litter. Two-way Anova and Tukey's multiple comparisons test performed. Adjusted *p-value for neonates = 0.9560, for pre-weaning = 0.7892*. **c-e,** Liver-to-body weight ratio, liver weight, and body weight measured in 26-day-old NICD^{venous} (n=6), APJ^{CreERT2} (n=5), and control (negative for CreERT2) littermate males (n=22). Mice are color-coded by litter. Two-way Anova and Tukey's multiple comparisons test performed. Adjusted *p-values* from left to right *for liver-to-body weight ratio = 0.0003, 0.0310; for liver weight = 0.0013, 0.0627; for body weight = 0.0033, 0.1299*. **f,** Quantification of the extent of venous endothelial expansion through GFP label coverage in 26-day-old APJ^{CreERT2};mTmG;NICD (NICD^{venous}, n=2 males, n=3 females) and APJ^{CreERT2};mTmG (control, n=2 males, n=2 females) mice. **g-i,** Liver-to-body weight ratio, liver weight, and body weight measured in 26-day-old NICD^{venous} (n=9), APJ^{CreERT2} (n=11), and control (negative for CreERT2) littermate females (n=22). Mice are color-coded by litter. Two-way Anova and Tukey's multiple comparisons test performed. Adjusted *p-values* from left to right *for liver-to-body weight ratio = 0.2064, 0.0163; for liver weight = 0.0014, 0.3513; for body weight = 0.0070, 0.9963*. **j,** Liver-to-body weight ratio in control littermate (mTmG or no transgene, n=10 at P26, n=7 at P56), APJ^{CreERT2} (n=7 at P26, n=3 at P56), and NICD (n=13 at P26, n=4 at P56) males at weaning age P26 and adulthood at 8 weeks. **k,** Quantification of lobule size measured as absolute area (left) and number of nuclei per lobule (right) in APJ^{CreERT2};mTmG;NICD (NICD^{venous}, n=5), APJ^{CreERT2} alone (n=2), and littermate control (n=5) males at 26 days. Black bars represent mean values. Ordinary one-way Anova and Tukey's multiple comparisons test were performed. From left to right, adjusted *p-values for lobule area = 0.2165, 0.0189, 0.3446*. From left to right, adjusted *p-values for number of nuclei = 0.1293, 0.0331, 0.7177*. **l,** Lethality in NICD^{venous} is shown as monitored percent mortality for all NICD^{venous} mice genotyped, compared to that of control littermates, for male and female mice. Males: Neonatal at P8, n=23 total pups, n=8 NICD^{venous}. Pre-wean at P14, n=13 total pups, n=3 NICD^{venous}. Post-wean at P26 and P34, n=46 total mice, n=8 NICD^{venous}. Adult at 8 weeks, n=2 total mice, n=0 NICD^{venous}. Females: Neonatal at P8, n=21 total pups, n=4 NICD^{venous}. Pre-wean at P14, n=7 total pups, n=1 NICD^{venous}. Post-wean at P26 and P34, n=50 total mice, n=12 NICD^{venous}. Adult at 8 weeks, n=7 total mice, n=0 NICD^{venous}. **m,** *In situ* hybridization on 14-day-old control (left) and NICD^{venous} (right) liver sections for *Wnt2* (top) and *Wnt9b* (bottom) along with the endothelial marker *Pecam*. Scale bars, 50 μm. **n,** Quantification of the number of lobules in 26-day-old control (n=7) and NICD^{venous} (n=5) male mice, mean values ± s.d. Unpaired t test, *p-value = 0.8274*. ns, not significant. PV, portal vessel. CV, central vein. Source data are provided in the Supp. Figures 5-7 Source Data file.

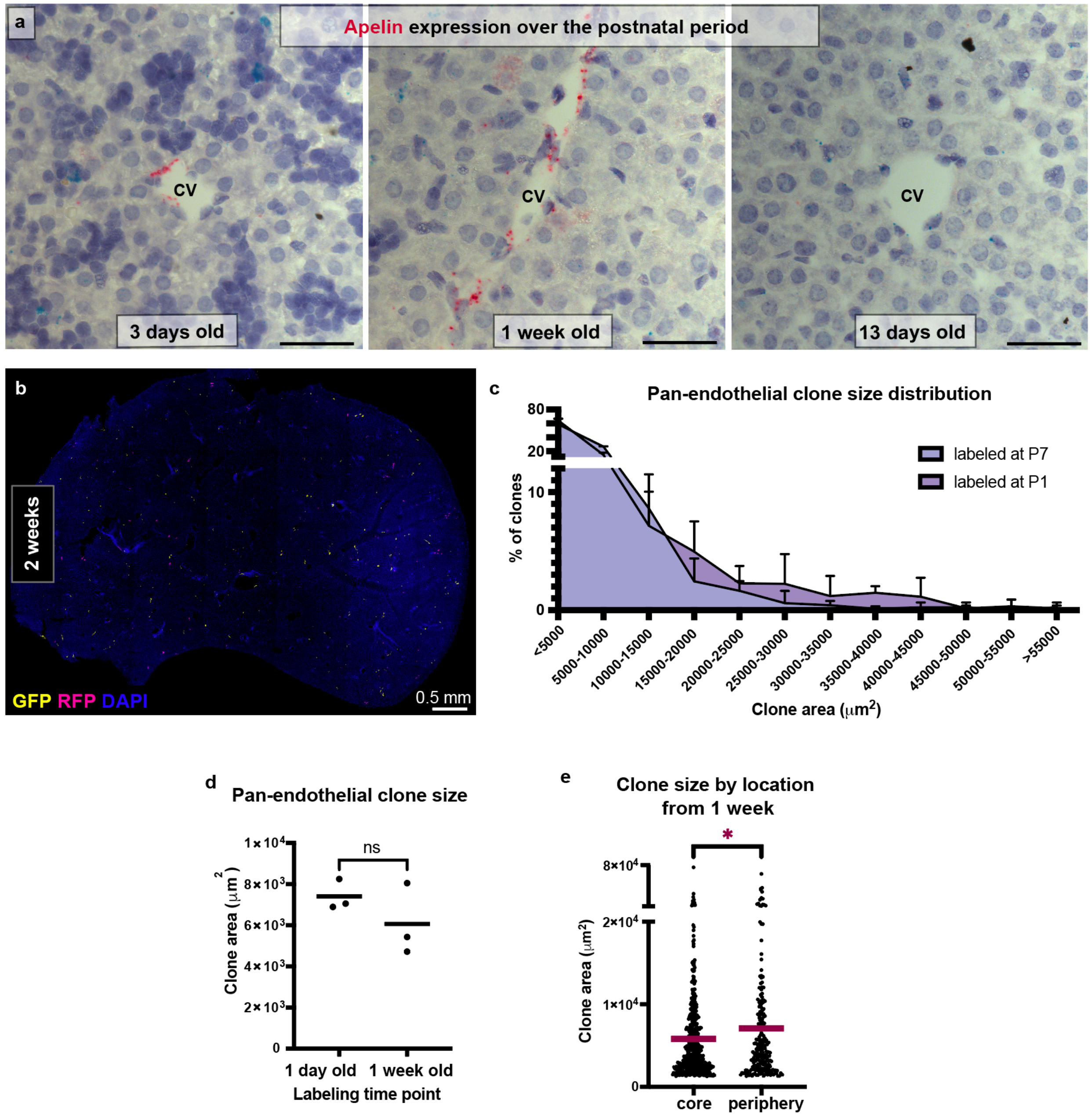

Extended Data Fig. 7 Endothelial expansion after the first neonatal week. a, *In situ* hybridization on 3-day-old, 1-week-old, and 13-day-old mouse livers for Apelin. Scale bars, 50 µm. **b,** VE-Cadherin^{CreERT2};Confetti thick liver section immunostained for GFP and RFP to mark the labeling events at 2 weeks following induction at 1 week. **c,** Distribution of endothelial clone size in 8-week-old VE-Cadherin^{CreERT2};Confetti livers comparing induction at 1 day versus 1 week, n=457 clones from 3 mice (induced at 1 day) and n=702 clones from 3 mice (induced at 1 week). Mean values + s.d. **d,** Comparison of average endothelial clone size per animal in 8-week-old mice that were traced from 1 day versus from 1 week, same dataset as Fig. 6i. n=457 clones from 3 mice (labeled at 1 day) and n=702 clones from 3 mice (labeled at 1 week). Black bars represent mean values. Unpaired t test, *p-value = 0.2912*. **e,** Quantification of clone size in the periphery compared to in the core from 8-week-old VE-Cadherin^{CreERT2};Confetti mice following induction at 1 week. n=473 (in the core) and n=229 (in

the periphery) clones were scored from 3 mice. Red bars represent mean values. Welch's t test, *p-value* = *0.0455*. DAPI in blue depicts nuclei. CV, central vein. Source data are provided in the Supp. Figures 5-7 Source Data file.
