# Supplementary material for: Vascularization of neonatal liver lobules presages adult liver size
Source: Nat Commun. 2025 Nov 13;16:9989. doi: 10.1038/s41467-025-64930-w (PMC12615705; doi:10.1038/s41467-025-64930-w)
Supplement: Supplementary file 3 — Description of Additional Supplementary Files [file 41467_2025_64930_MOESM3_ESM.pdf]

Description of Additional Supplementary Files

File Name: Supplementary Movie 1
Description: Animation of optical slices through light-sheet imaging of iDisco-cleared liver immunostained for EPCR to mark lobule boundaries.

File Name: Supplementary Movie 2
Description: High magnification taken from Supplementary Movie 1 zoomed in on a few lobules, showing vascular structures of the lobules, and pinpointing vein merging as the lobule boundaries disappear.

File Name: Supplementary Movie 3
Description: Animation of optical slices through light-sheet imaging of iDisco-cleared $Apelin^{mTmG}$ liver immunostained for GFP (lineage labeling) and EPCR (lobule boundaries).
